# Targeting Lp-PLA2 inhibits profibrotic monocyte-derived macrophages in silicosis through restoring cardiolipin-mediated mitophagy

**DOI:** 10.1038/s41423-025-01288-5

**Published:** 2025-05-19

**Authors:** Shifeng Li, Hong Xu, Shupeng Liu, Jinkun Hou, Yueyin Han, Chen Li, Yupeng Li, Gaigai Zheng, Zhongqiu Wei, Fang Yang, Shuwei Gao, Shiyao Wang, Jing Geng, Huaping Dai, Chen Wang

**Affiliations:** 1 National Center for Respiratory Medicine, State Key Laboratory of Respiratory Health and Multimorbidity, National Clinical Research Center for Respiratory Diseases, Institute of Respiratory Medicine, Chinese Academy of Medical Sciences, Department of Pulmonary and Critical Care Medicine, China-Japan Friendship Hospital, Beijing, China; 2 Health Science Center, Hebei Key Laboratory of Organ Fibrosis, North China University of Science and Technology, Tangshan, Hebei China; 3 School of Public Health, Hebei Key Laboratory of Organ Fibrosis, North China University of Science and Technology, Tangshan, Hebei China; 4 Peking Union Medical College, Beijing, China; 5 Immune Dysfunction and Pulmonary Fibrosis Joint Laboratory for Clinical Medicine, Capital Medical University, Beijing, China; 6 Second Affiliated Hospital of Harbin Medical University, Respiratory and Critical Care Medicine, Harbin, Heilongjiang China; 7 Basic Medical College, Hebei Key Laboratory of Organ Fibrosis, North China University of Science and Technology, Tangshan, Hebei China

**Keywords:** Lipoprotein-associated phospholipase A2, Cardiolipin, Mitophagy, Monocyte-derived macrophages, Pulmonary fibrosis, Immunogenetics, Alveolar macrophages

## Abstract

Monocyte-derived macrophages (MoMacs) are the most important effector cells that cause pulmonary fibrosis. However, the characteristics of MoMac differentiation in silicosis and the mechanisms by which MoMacs affect the progression of pulmonary fibrosis remain unclear. Integration of single-cell and spatial transcriptomic analyses revealed that the silicosis niche was occupied by a subset of MoMacs, identified as Spp1^hi^Macs, which remain in an immature transitional state of differentiation during silicosis. This study investigated the mechanistic foundations of mitochondrial damage induced by the lipoprotein-associated phospholipase A2 (Lp-PLA2, encoded by *Pla2g7*)–acyl-CoA:lysocardiolipin acyltransferase-1 (ALCAT1)–cardiolipin (CL) signaling pathway, which interferes with Spp1^hi^Mac differentiation. We demonstrated that in SiO_2_-induced MoMacs, Lp-PLA2 induces abnormal CL acylation through the activation of ALCAT1, resulting in impaired mitochondrial localization of PINK1 and LC3B and mitochondrial autophagy defects. Simultaneously, lysosomal dysfunction causes the release of the lysosomal protein cathepsin B into the cytoplasm, which involves M1 and M2 macrophage polarization and the activation of proinflammatory and profibrotic pathways. Furthermore, we assessed the efficacy of the Lp-PLA2 inhibitor darapladib in ameliorating silica-induced pulmonary fibrosis in a murine model. Our findings enhance our understanding of silicosis pathogenesis and offer promising opportunities for developing targeted therapies to mitigate fibrotic progression and maintain lung function in affected individuals.

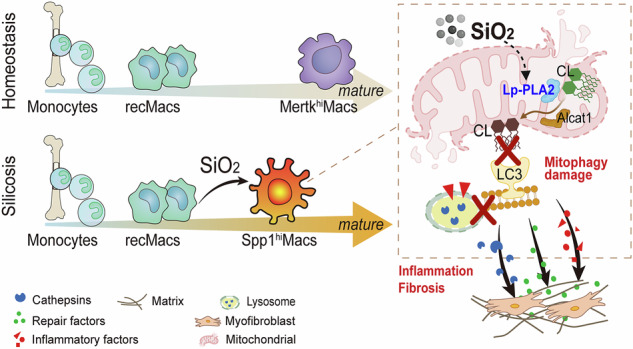

## Introduction

Silicosis (pneumoconiosis) is a severe, progressive, and incurable fibrotic lung disease caused by the inhalation of crystalline silica particles (SiO_2_) generated in industries such as quarrying and coal mining. This condition can lead to respiratory failure [1–3]. Currently, no clear strategies exist for early diagnosis, progress monitoring, or efficacious treatment [2–5].

Macrophages, as primary responders to silica exposure, play a crucial role in facilitating the fibrotic process of silicosis [6–8]. Our previous studies demonstrated that typical pathological changes in silicosis, including initial increases in small cellular nodules and the development of fibrotic cellular nodules in advanced stages, correlate with the functional regulation of macrophages [6–9]. Macrophages facilitate inflammatory processes through M1 polarization and drive fibrotic repair and pulmonary fibrosis through M2 polarization [8, 10]. Recent studies have identified specific subsets of macrophages, highlighting the complex heterogeneity within this cell population. The cellular origin and anatomical localization are key factors influencing macrophage diversity, which can be divided into tissue-resident alveolar macrophages (TRAMs) and pulmonary interstitial macrophages (IntMacs), also known as monocyte-derived macrophages (MoMacs) [11–13]. TRAMs, which originate from the embryonic mesoderm, are responsible for maintaining lung homeostasis and responding to injury through efficient efferocytosis [14]. Under prolonged or intense stimulation, TRAMs become susceptible to damage and depletion, leading to their replacement by MoMacs migrating from the region between the alveolar and vascular endothelial layers [11, 13, 15]. Unlike TRAMs, TRAMs exhibit greater phenotypic plasticity and polarization capacity, allowing them to dynamically adjust their epigenetic landscape, gene expression profiles, and metabolic states in response to microenvironmental stimuli [11, 12, 16]. This inherent adaptability enables MoMacs to develop unique phenotypes suited to specific environmental challenges, significantly impacting tissue homeostasis and repair processes. However, their phenotypic plasticity also makes them susceptible to modification within fibrotic environments, thereby facilitating the progression of pulmonary fibrosis [12]. Increasing evidence underscores the importance of MoMacs as critical cellular subsets in the pathogenesis of pulmonary fibrosis. Targeting monocyte recruitment and activation represents a promising therapeutic approach [13, 15]. However, therapeutic interventions aimed at altering monocyte populations may have unintended consequences, particularly in organs reliant on monocytes for sustaining macrophage turnover rates, such as the intestine [17]. Therefore, unraveling the intricate phenotypic alterations of MoMacs upon infiltration of target organs during silicosis progression and elucidating the underlying molecular mechanisms governing their cellular phenotypes are critically important.

The lung is a highly phospholipid-rich organ that continuously produces, secretes, and recycles the primary components of surfactants, which are mainly phospholipids. Alveolar macrophages are essential for the clearance of oxidized lipids within this cycle [14]. Therefore, pulmonary diseases frequently correlate with disturbances in phospholipid metabolism, including lipid peroxidation [18]. This mechanism induces lipoprotein-associated phospholipase A2 (Lp-PLA2, encoded by *Pla2g7*) expression, leading to phospholipid degradation [19, 20]. We previously revealed elevated levels of phospholipid metabolism products in silicosis-associated fibrosis [21]. Additionally, lipid mediators, including pro-inflammatory arachidonic acid (AA), prostaglandin E2 (PGE2), and pro-resolving protectin D1 (PD1), influence MoMac maturation, polarization, and their regulation of inflammation persistence or resolution [22, 23]. Furthermore, the interplay between lipid metabolism and mitochondrial function in macrophages can affect macrophage polarization [24]. However, the regulatory effects of lipid mediators on macrophage plasticity and MoMac differentiation are unclear. Consequently, a detailed characterization of the metabolic traits of the macrophage subsets involved in fibrosis and their correlation with the macrophage phenotype and differentiation status may yield essential insights into the mechanisms underlying silicosis development and progression.

This study conducted a comprehensive investigation of silicosis-related pulmonary fibrosis through single-cell sequencing (scRNA-seq) of silica-exposed mice at various time points to delineate their evolutionary trajectories. Furthermore, spatial transcriptomic sequencing (ST-seq) was utilized to reconstruct niche information, further facilitating the elucidation of key targets involved in silicosis-related fibrosis. Our study revealed the transitional immature differentiation state of inflammatory alveolar macrophages termed fibrotic macrophages (Spp1^hi^Macs), which originate from monocytes in response to silica-induced injury. This study revealed that Lp-PLA2 regulates cardiolipin (CL) metabolism and induces mitophagy disorders, which underlie the impaired differentiation of Spp1^hi^Macs and persistent chronic inflammation in silicosis. Our findings provide new insights into silicosis pathophysiology and pave the way for the development of targeted therapeutic strategies to mitigate the severe effects of silicosis.

## Results

### scRNA-Seq and ST-seq profiling map heterogeneity of macrophage clusters and tissue niches in silicotic lungs

**Fig. 1:**
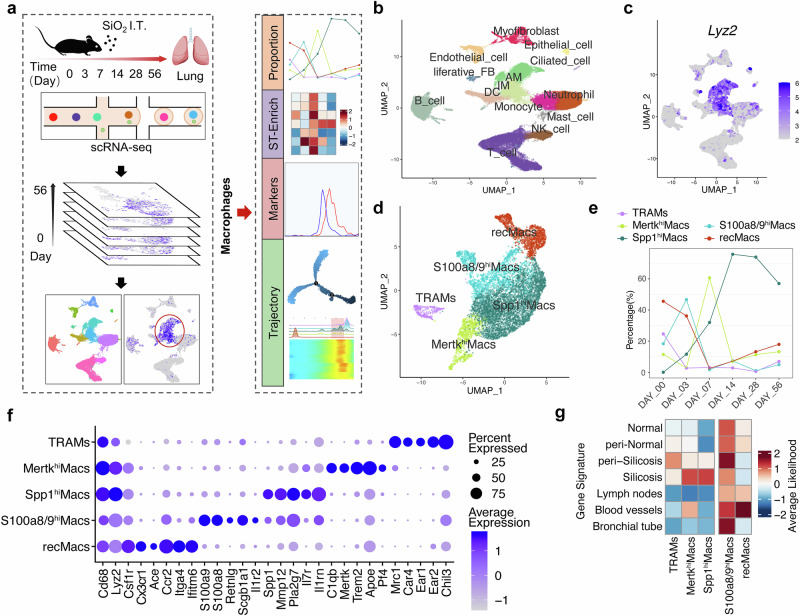
Cellular composition and macrophage subclusters in silicotic lungs. **a** Flow chart of the study design and analysis. (**b**) UMAP plots showing the identified cell types annotated by known cell biomarkers (Supplementary Fig. S1). **c** Macrophage clusters (including AM and IM in (**b**)) were identified via *Lyz2*, as shown on UMAP feature plots, and the intensity of expression is indicated by purple color. **d** Clusters of cells expressing *Lyz2* were subsets from the main dataset and reclustered, revealing five subclusters, including TRAMs, Mertk^hi^Macs, Spp1^hi^Macs, S100a8/9^hi^Macs, and recMacs. **e** The percentage of macrophage subclusters in lung tissue as silicosis progresses. **f** Gene expression of key markers in each macrophage subcluster. **g** Predicted distribution of five macrophage subclusters in pulmonary niches

We performed scRNA-seq on lung cells isolated from silicotic mice subjected to bronchial instillation of SiO_2_ for 3, 7, 14, 28, and 56 days or PBS (0 days). Bioinformatics tools were utilized to analyze the subpopulation characteristics, proportion changes, and differentiation trajectories of macrophages (Fig. 1a), enabling us to understand the phenotypic reshaping characteristics of monocytes and macrophages during silicosis progression and investigate the key mechanisms influencing cell phenotypes. After quality control and filtering, the analyzed dataset comprised 50,195 cells. The cell clusters were manually annotated via previously described cell markers (Fig. 1b and Supplementary Fig. S1). A focused analysis of macrophage populations identified five subclusters: TRAMs, Mertk^hi^Macs, Spp1^hi^Macs, S100a8/9^hi^Macs, and recMacs (Fig. 1c–e), distinguished by the markers illustrated in Fig. 1f. The characteristic genes of TRAMs are associated with the normal homeostatic function of alveolar macrophages. RecMacs and S100a8/9^hi^Macs express genes associated with the inflammatory response, cytokine synthesis, and the innate immune response. The characteristic genes of Mertk^hi^Macs are associated with phagocytosis, collagen degradation, and negative regulation of the inflammatory signals interleukin 1β (IL-1β) and tumor necrosis factor (TNF). In contrast, Spp1^hi^Macs presented a high abundance of profibrotic genes associated primarily with injury and inflammatory responses, IL-1β secretion, collagen synthesis, the transforming growth factor-β (TGF-β) signaling pathway, and smooth muscle cell proliferation (Fig. 1f and Supplementary Fig. S2).

We observed a significant decrease in the proportion of TRAMs during silicosis progression. The proportion of recMacs and S100a8/9^hi^Macs markedly decreased on day 3, whereas the proportion of Mertk^hi^Macs decreased on day 7 after SiO_2_ exposure. However, the proportion of Spp1^hi^Macs consistently increased with disease progression (Fig. 1e). Additionally, we delineated the spatial distribution of the macrophage subclusters by correlating the characteristic genes of the macrophage subclusters with the niche-specific genes identified through ST-seq (Fig. 1g and Supplementary Fig. S3). We found that TRAMs were scattered throughout fibrotic niches, that RecMacs were primarily distributed within blood vessels, that S100a8/9^hi^Macs were diffusively distributed in the lung tissue and vasculature, that Mertk^hi^Macs were primarily concentrated in and around fibrotic areas, and that Spp1^hi^Macs were confined to fibrotic niches (Fig. 1g). Considering the proportion and localization characteristics of macrophage subsets during silicosis, our subsequent study focused on the Spp1^hi^Mac subsets.

### Spp1^hi^Macs are in an immature differentiation state and acquire profibrotic potential during silicosis progression

**Fig. 2:**
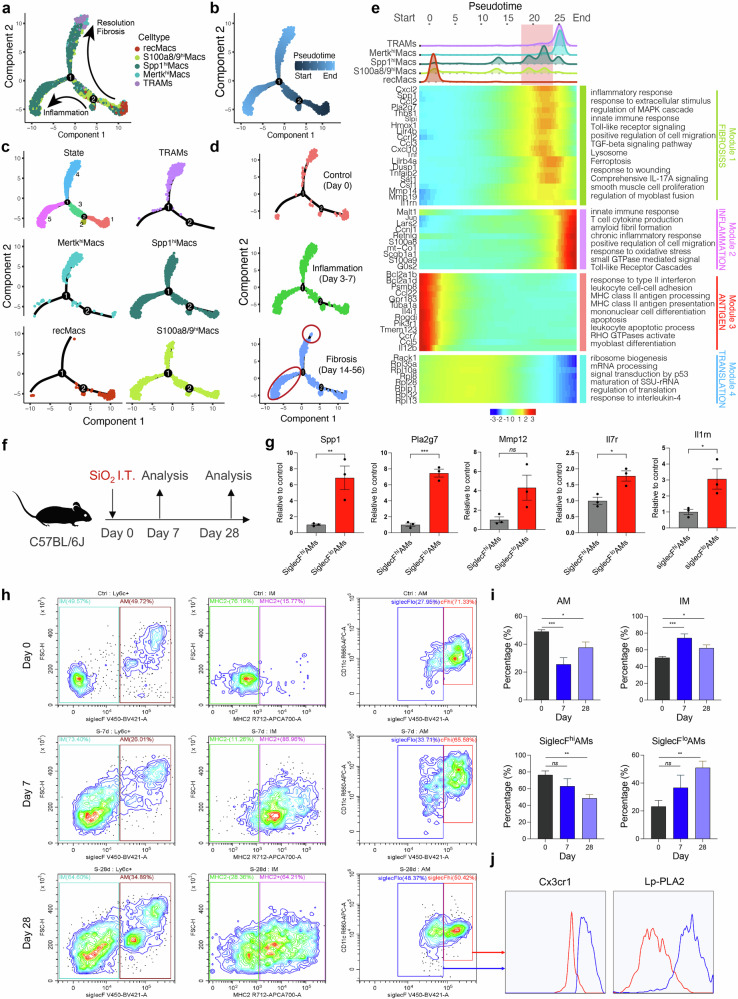
Trajectory analysis of macrophage subclusters in silicosis revealed distinct features. Differentiation trajectory of macrophages in all lung samples with points colored by **a** macrophage subcluster and **b** pseudotime. **c** Differentiation trajectory of each macrophage subcluster. **d** Differentiation trajectory of macrophages in the control (0 d), inflammation (3 d and 7 d), and fibrosis (14 d, 28 d, and 56 d) groups. **e** Pseudoheatmap showing the various genes involved in the differentiation process of macrophages, which were clustered into 4 clusters related to fibrosis, inflammation, antigens, and translation. **f** Schematic diagram of the silicosis mouse model. **g** mRNA expression levels of *Spp1, Mmp12, Pla2g7, Il17r*, and *Il1rn* in AM subclusters sorted from silicosis and control mouse lung tissue, *n* = 3 per group. **h**, **i** Proportion of IM subclusters and AM subclusters in silicosis mouse lungs analyzed by FC. *n* = 3 per group. **j** Fluorescence of Cx3cr1 and Lp-PLA2 between SiglecF^lo^AMs and SiglecF^hi^AMs. The data are presented as the means ± SDs; *p < 0.05, **p < 0.01, ***p < 0.001, ^ns^ *p* ≥ 0.05

Pseudotime analysis of macrophages from all the samples revealed that RecMacs were in the early differentiation state. Conversely, TRAMs were in the terminal differentiation state (Fig. 2a–c). The differentiation pathway of Mertk^hi^Macs is similar to that of TRAMs, indicating a relatively advanced differentiation state of Mertk^hi^Macs. We unexpectedly observed a significant overlap in the differentiation trajectory between Spp1^hi^Macs and S100a8/9^hi^Macs, exhibiting a flexible and bidirectional differentiation pattern. However, only Spp1^hi^Macs, rather than S100a8/9^hi^Macs, exhibited a persistent increase, indicating that lung tissue remodeled Spp1^hi^Macs to a greater extent. In addition, Spp1^hi^Macs presented a reduced degree of differentiation along the same branches as Mertk^hi^Macs or TRAMs did (Fig. 2c, d). Next, we used fate-mapping tools to identify key differential genes involved in the trajectory of macrophage alterations in silicosis. Notably, a fibrosis-associated gene module emerged before the end state peak, with the peak gene expression within this module aligning with that of Spp1^hi^Macs (Fig. 2e, red background). These findings reveal that, during silicosis, fibrosis-associated gene modules exhibit sustained high expression during the monocyte-to-AM transition, potentially representing a crucial point affecting AM maturation.

Flow cytometry revealed that during the 7-day inflammatory phase of silicosis, the proportion of AMs decreased, followed by an increase during the 28-day fibrotic phase, whereas the interstitial macrophage (IM) proportion exhibited an inverse trend relative to alveolar macrophages (Fig. 2f–i and Supplementary Fig. S4). It is believed that differences in Siglec F expression levels facilitate the differentiation of MoMacs and TRAMs during injury [25]. Further analysis of AM subpopulations was performed using Siglec F as a marker to differentiate the maturation level of monocyte-derived alveolar macrophages [15]. The level of the monocyte-specific gene Cx3cr1 was greater in SiglecF^lo^AMs than in SiglecF^hi^AMs, indicating the monocyte origin of SiglecF^lo^AMs (Fig. 2j). Notably, the expression levels of Spp1^hi^Mac marker genes (*Spp1, Mmp12, Pla2g7, Il7r, and Il1rn*) were higher in SiglecF^lo^AMs than in SiglecF^hi^AMs (Fig. 2g), suggesting that Spp1^hi^Macs share similar properties and exhibit immature differentiation characteristics such as SiglecF^lo^AMs. The proportion of SiglecF^hi^AMs decreased, whereas that of SiglecF^lo^AMs increased during silicosis progression (Fig. 2h, i), which was consistent with the trend observed for TRAMs and Spp^hi^Macs via scRNA-seq analysis.

### Sustained expression of *Pla2g7* is important for the profibrotic mechanism of Spp1^hi^Macs in silicotic mice

**Fig. 3:**
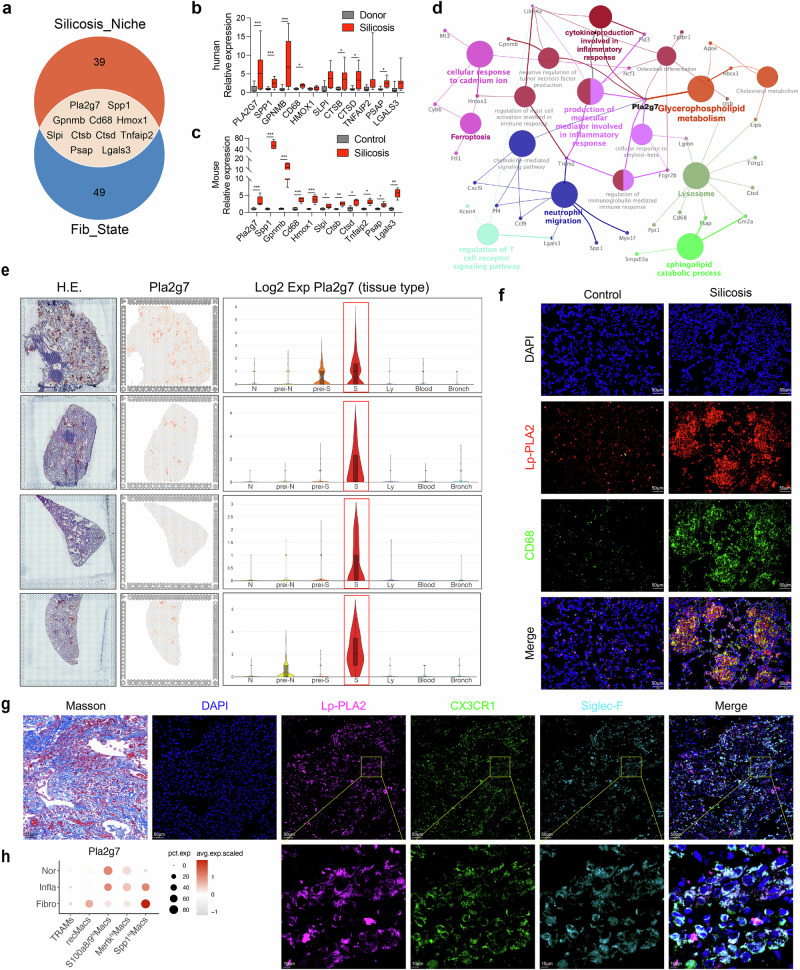
Expression of the key gene *Pla2g7* in macrophages from silicosis lung tissue. **a** Venn diagram showing the intersection of the characteristic genes of the silicosis niche detected by ST-seq with the characteristics of the fibrosis module in pseudotime and the key fibrotic genes. **b** mRNA expression of key fibrotic genes in lung tissue from silicosis patients and donors. *n* = 5 per group. **c** mRNA expression of key fibrotic genes in lung tissue from control and silicosis mice. *n* = 5 per group. **d** Gene interaction network and functional enrichment analysis of the genes characteristic of the silicosis niche detected by ST-seq. **e** Expression of Pla2g7 in different lung tissue niches detected by ST-seq. **f** Codistribution of Lp-PLA2 and CD68 in the lung tissues of silicosis model mice, as determined by immunofluorescence staining; scale bar = 50 μm. **g** Codistribution of Lp-PLA2, Cx3cr1, and SiglecF in the lung tissues of silicosis patients, as determined by immunofluorescence staining; scale bar = 50 μm; zoom bar = 10 μm. **h** Expression of *Pla2g7* in macrophage subsets during the normal fibrotic (0 days), inflammatory (3–7 days) and fibrotic (14–56 days) stages, as detected via scRNA-seq. The data are presented as the means ± SDs; *p < 0.05, **p < 0.01, ***p < 0.001

We integrated data on silicosis fibrotic niches with macrophage differentiation data by intersecting the top 50 feature genes of fibrotic niches identified through ST-seq with the 60 feature genes indicative of fibrosis initiation in the trajectory of Spp1^hi^Macs, resulting in 11 key genes (*Pla2g7, Spp1, Gpnmb, Cd68, Hmox1, Slpi, Ctsb, Ctsd, Tnfaip2, Psap*, and *Lgals3*) (Fig. 3a). The expression of these genes in the lung tissues of patients with silicosis, donor lung transplant samples, and control and silicosis mouse models was validated, and the results revealed consistent expression trends across different species, with upregulation during fibrosis progression. (Fig. 3b, c). *Pla2g7* expression was specific to the fibrotic niche of silicosis, as demonstrated by the ST-seq data (Fig. 3e). Additionally, *Spp1* showed collinearity with *Pla2g7* in macrophages (Supplementary Fig. S5a, b). Protein‒protein interaction (PPI) network analysis of highly expressed genes in the fibrotic niche identified *Pla2g7* as a hub gene enriched in functions associated with lipid metabolism and inflammation (Fig. 3d). Immunofluorescence staining revealed that the *Pla2g7*-encoded protein Lp-PLA2 was highly expressed in fibrotic regions of silicotic mice and coexpressed with the macrophage marker CD68. Similarly, Lp-PLA2 colocalizes with Cx3cr1 and Siglec F in the fibrotic lung tissue of silicotic patients (Fig. 3f, g). Flow cytometry revealed higher Lp-PLA2 expression levels in SiglecF^lo^AMs than in SiglecF^hi^AMs (Fig. 2j). Sc-RNA-seq analysis revealed the progressive upregulation of the *Pla2g7* gene in Spp1^hi^Macs during the transition from a normal state to an inflammatory state to a fibrotic state (Fig. 3h). These findings highlight the crucial role of *Pla2g7* in modulating macrophage differentiation and silicosis-related fibrosis.

**Fig. 4:**
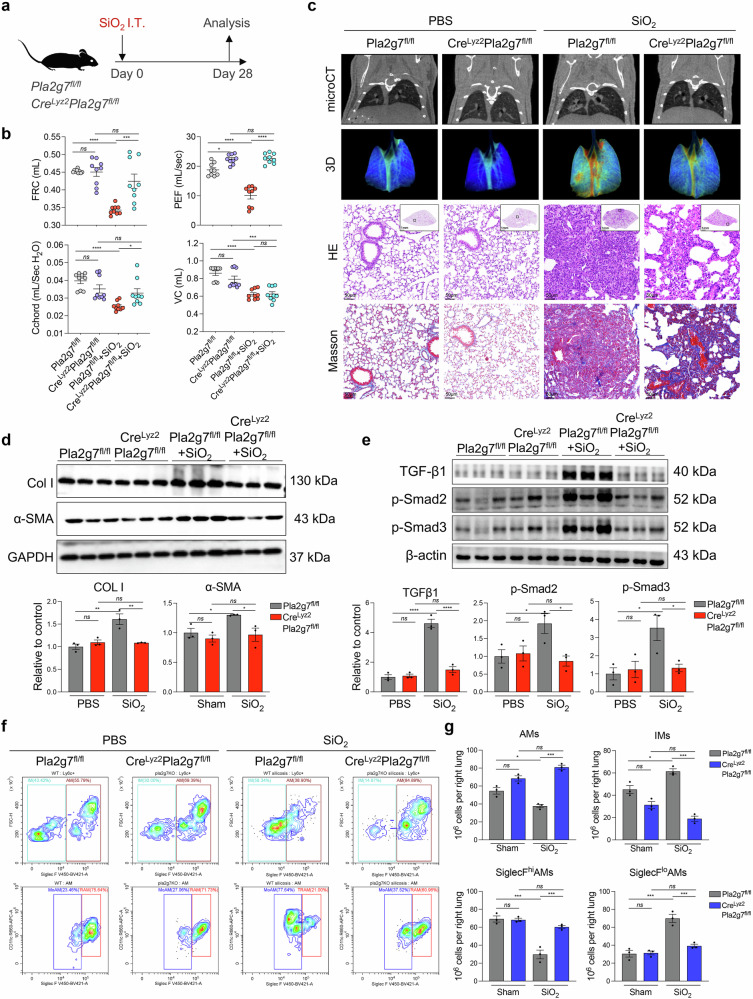
Effects of macrophage-specific *Pla2g7* knockout on silicosis fibrosis and macrophage subsets in mice. **a** Cre^lyz2^Pla2g7^fl/fl^ and Pla2g7^fl/fl^ mice were treated with a single intratracheal administration of silica to induce silicosis or with PBS as a control, and the mice were sacrificed on the 28th day after administration. **b** Lung function of the FRCs, PEFs, Cchords, and VCs of the mice in different experimental groups. *n* = 9 per group. **c** Micro-CT image, lung tissue oxygenation, HE, and Masson staining of Cre^lyz2^Pla2g7^fl/fl^ and Pla2g7^fl/fl^ mice stimulated with SiO_2_. Scale bar = 1 mm; zoom bar = 50 μm. **d** The protein expression of α-SMA and Col I in the lung tissue of Cre^lyz2^Pla2g7^fl/fl^ and Pla2g7^fl/fl^ mice stimulated with SiO_2_. *n* = 3 per group. **e** The protein expression of TGF-β1, p-Smad2, and p-Smad3 in the lung tissue of Cre^lyz2^Pla2g7^fl/fl^ and Pla2g7^fl/fl^ mice stimulated with SiO_2_. *n* = 3 per group. **f**, **g** Proportion of macrophage subsets in mice detected by FC; *n* = 3 per group. The data are presented as the means ± SDs; *p < 0.05, **p < 0.01, ***p < 0.001, ^ns^ *p* ≥ 0.05

Furthermore, we generated genetically modified mice by deleting *Pla2g7* from macrophages via Cre drivers (Cre^Lyz2^Pla2g7^flox/flox^) to specifically target macrophages, and their Pla2g7^flox/flox^ littermates served as controls (Fig. 4a and Supplementary Fig. S6). Cre^Lyz2^Pla2g7^flox/flox^ mice and Pla2g7^flox/flox^ mice were intratracheally administered SiO_2_ to assess the severity of pulmonary fibrosis and the proportion of recruited SiglecF^lo^AM. Pulmonary function tests and microcomputed tomography imaging revealed that, compared with their Pla2g7^flox/flox^ counterparts, Cre^Lyz2^Pla2g7^flox/flox^ mice presented increased functional residual capacity, elevated peak expiratory flow, and increased pulmonary oxygenation. (Fig. 4b, c). Micro-CT, HE and Masson’s trichrome staining revealed reduced fibrotic nodules, inflammatory infiltrates and collagen deposition (Fig. 4c), along with decreased expression of fibrotic markers, including alpha-smooth muscle actin (α-SMA), collagen I (COL I), and TGF-β-smad2/3 pathway proteins (Fig. 4d, e). Flow cytometry analysis of lung-infiltrating macrophages revealed a reduction in profibrotic SiglecF^lo^AM subsets in Cre^Lyz2^Pla2g7^flox/flox^ mice compared with those in Pla2g7^flox/flox^ mice, indicating a crucial role for *Pla2g7* in modulating macrophage function and its contribution to silicosis-induced fibrosis (Fig. 4f, g).

### *Pla2g7* deficiency inhibits macrophage polarization in silicotic mice and SiO_2_-induced MoMacs

**Fig. 5:**
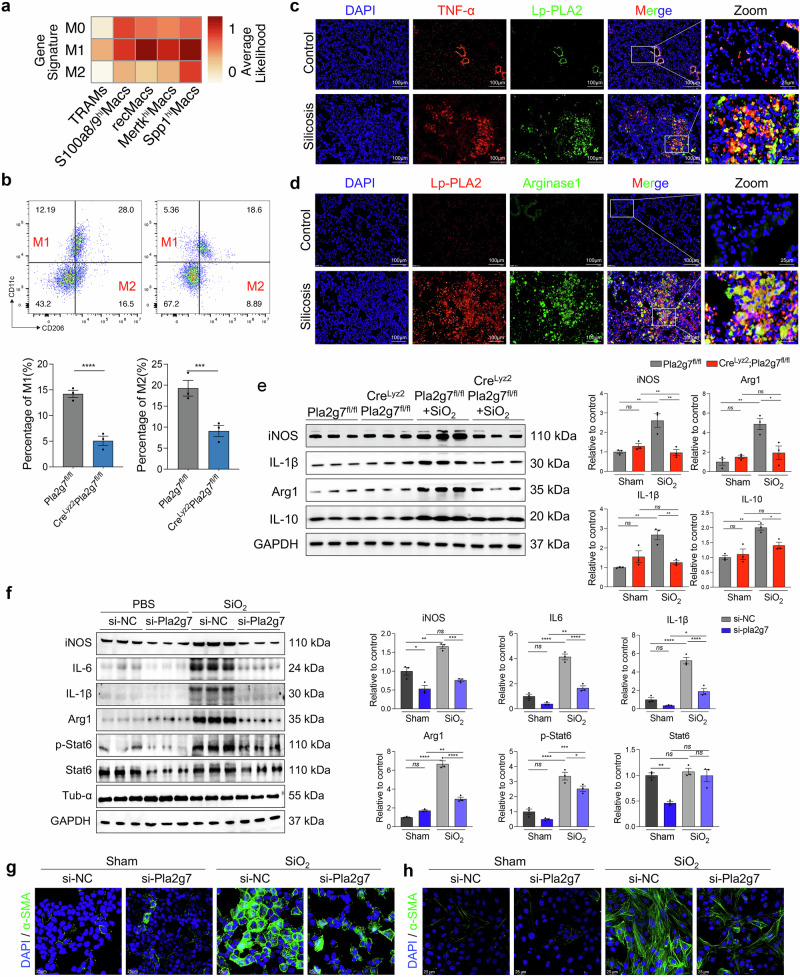
Loss of *Pla2g7* attenuated M1 and M2 polarization in macrophages. **a** Classification of macrophage subsets by M0/M1/M2-like gene signatures detected by scRNA-seq. **b** FC analysis of M1 and M2 macrophages in the lung tissue of Cre^lyz2^Pla2g7^fl/fl^ and Pla2g7^fl/fl^ mice stimulated with SiO_2_, *n* = 3. **c**, **d** Colocalization of Lp-PLA2 with TNF-α and Lp-PLA2 with Arg1 in the lung tissues of silicosis mice as detected by immunofluorescence; scale bar = 100 μm. **e** Protein expression of iNOS, IL-1β, Arg 1, and IL-10 in the lung tissue of Cre^lyz2^Pla2g7^fl/fl^ and control Pla2g7^fl/fl^ mice treated with SiO_2_; *n* = 3. **f** Western blot analysis of iNOS, IL-6, IL-1β, Arg 1, p-Stat6, and Stat6 expression in SiO_2_-stimulated BMDMs transfected with scrambled (si-NC) or *Pla2g7* siRNA (si-*Pla2g7*), *n* = 3. **g**, **h** Expression of α-SMA in MLE12 alveolar epithelial cells and primary fibroblasts cocultured with SiO_2_-stimulated BMDMs transfected with si-NC or si-*Pla2g7*; scale bar = 25 μm. The data are presented as the means ± SDs; *p < 0.05, **p < 0.01, ***p < 0.001, ^ns^ *p* ≥ 0.05

Polarization represents macrophage activation and functional execution, with M1 and M2 polarization contributing to the development of silicosis [8]. ScRNA-seq analysis revealed that, with the exception of TRAMs, other macrophage subsets are highly correlated with M1 polarization. However, compared with other macrophage subsets, Spp1hi Macs Spp1^hi^Macs presented the strongest correlation with M2 polarization (Fig. 5a). The immunostaining results revealed strong coexpression of Lp-PLA2 with tumor necrosis factor-α (TNF-α) and arginase 1 (Αrg1), which are markers of M1 and M2 polarization, in fibrotic mouse lung tissues (Fig. 5c, d). Flow cytometry analysis of lung macrophages revealed a significantly lower percentage of lung M1 (CD11c^+^ CD206^-^) and M2 macrophages (CD11c^-^ CD206^+^) in Cre^Lyz2^Pla2g7^fl/fl^ mice than in Pla2g7^fl/fl^ mice after SiO_2_ induction (Fig. 5b). Consistently, western blot analysis confirmed a significant reduction in the expression of inducible nitric oxide synthase (iNOS), IL-1β, Arg1 and IL-10 in the lungs of Cre^Lyz2^Pla2g7^fl/fl^ mice (Fig. 5e).

Additionally, the expression levels of iNOS, IL-1β, Arg1, and IL-10 and the phosphorylation of Stat6 were significantly increased in SiO_2_-induced mouse bone marrow-derived macrophages (BMDMs), which were inhibited by *Pla2g7* silencing (Fig. 5f). However, OE-*Pla2g7* collaborated with SiO_2_ in the MoMac cell line RAW264.7 to promote M1 and M2 polarization (Supplementary Fig. S7a–d). Furthermore, SiO_2_-stimulated BMDMs and OE-*Pla2g7*-transfected RAW264.7 cells induced a myofibroblast phenotype in cocultured MLE12 cells (through epithelial‒mesenchymal transition (EMT)) and mouse primary fibroblasts (through fibroblast‒myofibroblast transition (FMT)), as evidenced by increased expression of α-SMA, whereas BMDMs with *Pla2g7* silencing inhibited EMT or FMT (Fig. 5g–h, Supplementary Fig. S7e).

### Lp-PLA2 is translocated to the mitochondria in silica-induced MoMacs and promotes CL remodeling, leading to mitochondrial dysfunction

**Fig. 6:**
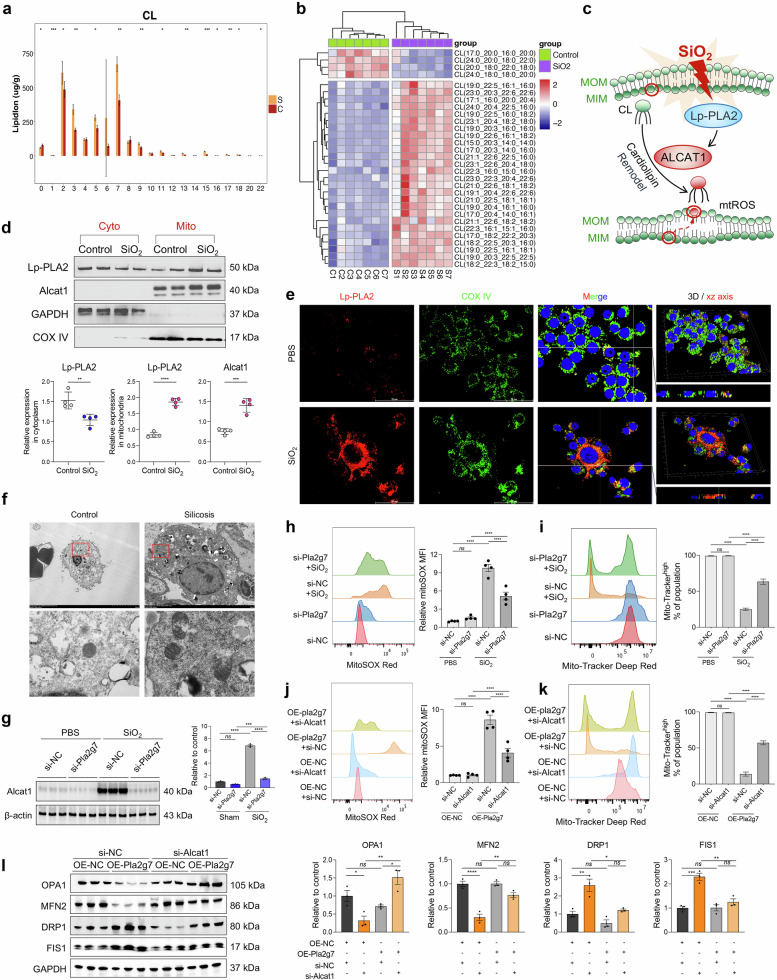
Effect of the Lp-PLA2-cardiolipin (CL) pathway on SiO_2_-induced mitochondrial damage in macrophages. **a** The saturation degree of different carbon units in the CL. **b** Heatmap of CL acylation levels. **c** Schematic diagram of the Lp-PLA2-Cl-mediated mitochondrial damage model. **d** Expression of Lp-PLA2 and ALCAT1 in the mitochondria and cytoplasm of SiO_2_-induced RAW264.7 cells, *n* = 4. **e** Colocalization of Lp-PLA2 and the mitochondrial marker protein COX IV detected by immunofluorescence. **f** Mitochondrial damage in the lung macrophages of control and silicosis mice was detected via TEM. **g** Western blot analysis of ALCAT1 expression in SiO_2_-induced BMDMs transfected with si-NC or si-*Pla2g7*; *n* = 3. **h** The mitoSOX level in macrophages treated with SiO_2_ and si-*Pla2g7*; *n* = 4. **i** The mitochondrial membrane potential of macrophages treated with SiO_2_ or si-*Pla2g7*; *n* = 4. **j** MitoSOX levels in macrophages treated with OE-*Pla2g7* or si-*Alcat1*, *n* = 4. **k** Mitochondrial membrane potential of macrophages treated with OE-*Pla2g7* or si-*Alcat1*, *n* = 4. **l** Western blot analysis of OPA1, MFN2, DRP1, and FIS1 expression in macrophages transfected with OE-*Pla2g7* or si-*Alcat1*, *n* = 3. The data are presented as the means ± SDs; *p < 0.05, **p < 0.01, ***p < 0.001, ^ns^ *p* ≥ 0.05

Lp-PLA2 functions as a phospholipase enzyme that regulates inflammation by catalyzing lipid metabolism [20, 26, 27]. ST niche enrichment analysis revealed that lipid metabolism pathways were significantly enriched in the pulmonary fibrotic niche (Fig. 3c and Supplementary Fig. S5c). Increased numbers of macrophage subsets in the alveolar lavage fluid of silicosis patients also resulted in abnormal lipid metabolism (Supplementary Fig. S8a–d). Subsequent lipidomic analysis of macrophages isolated from silicosis tissues revealed significant abnormalities in CL metabolism, characterized by elevated acylation and unsaturation levels (Fig. 6a, b and Supplementary Fig. S8e, f). CL is an essential lipid for maintaining mitochondrial homeostasis. The abnormal acylation and desaturation of CL by acyl-CoA:lysocardiolipin acyltransferase-1 (ALCAT1) renders CL more vulnerable to oxidation [28]. Lp-PLA2 acts as a crucial enzyme that catalyzes the oxidation and hydrolysis of CL, suggesting that SiO_2_-induced phenotypic alterations in macrophages may involve the regulatory effects of Lp-PLA2 on the ALCAT1-CL metabolic pathway and mitochondrial impairment.

Western blot analyses revealed the upregulation of Lp-PLA2 and ALCAT1 expression within the mitochondria of SiO_2_-induced macrophages (Fig. 6d). The immunostaining results revealed the colocalization of Lp-PLA2 and the mitochondrial marker COX IV (Fig. 6e). Transmission electron microscopy (TEM) revealed that the mitochondria of AMs from control mice were normal, whereas those from silicotic mice were swollen and vacuolated (Fig. 6f). However, silencing *Pla2g7* inhibited the increase in ALCAT1 protein levels (Fig. 6g) while maintaining the mitochondrial membrane potential and decreasing mitochondrial reactive oxygen species (mtROS) levels in SiO_2_-induced macrophages (Fig. 6h, i). *Pla2g7* overexpression diminished the mitochondrial membrane potential and increased mtROS levels, which could be restored through si-*Alcat1* transfection and treatment with the CL peroxidase inhibitor SS-31 (Fig. 6j, k and Supplementary Fig. S8g). Moreover, *Pla2g7*-overexpressing macrophages presented decreased expression of the mitochondrial fusion-related proteins optic atrophy 1 (OPA1) and mitofusin-2 (MFN2) and increased expression of the mitochondrial fission-related proteins dynamin-related protein 1 (DRP1) and fission protein 1 (FIS1), which were mitigated by silencing *Alcat1* (Fig. 6l).

### The Lp-PLA2-ALCAT1-CL pathway blunts LC3B and PINK1 binding to mitochondria, affects mitophagic flux and mediates pyroptosis in silica-induced macrophages

**Fig. 7:**
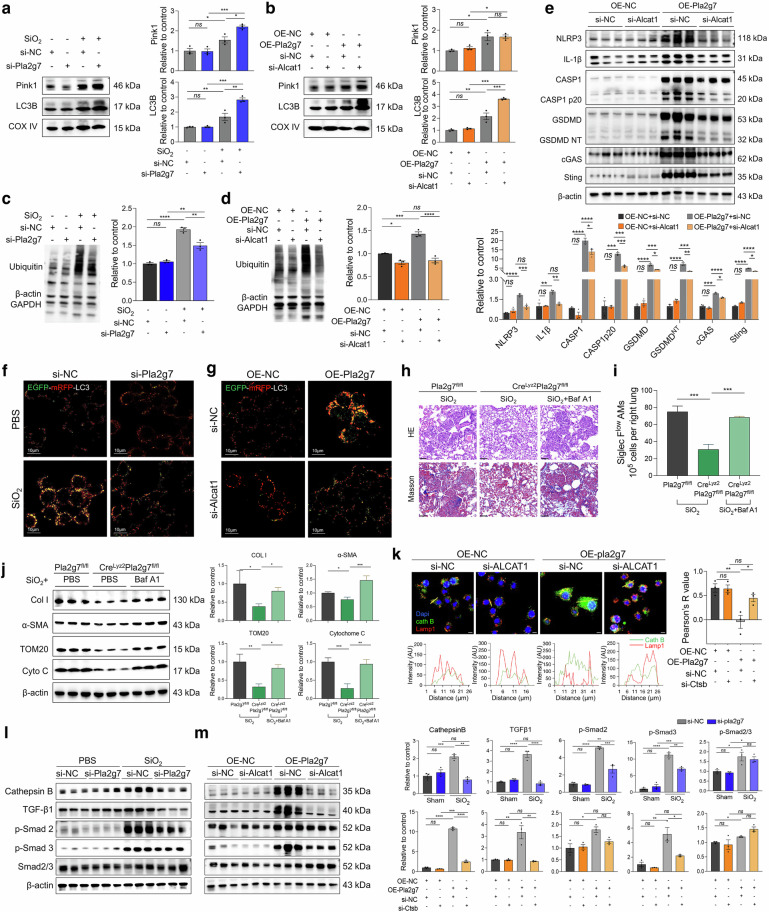
The Lp-PLA2-CL pathway induced mitophagy dysfunction in SiO_2_-induced macrophages and in SiglecF^lo^AM. **a** Protein expression of Pink1 and LC3B in the mitochondria of RAW264.7 cells treated with SiO_2_ and si-*Pla2g7*; *n* = 3. **b** Pink1 and LC3B expression in the mitochondria of macrophages treated with OE-*Pla2g7* or si-*Alcat1*; *n* = 3. **c** Ubiquitination levels of proteins in RAW264.7 cells treated with SiO_2_ and si-*Pla2g7*, *n* = 3. **d** Ubiquitination levels of proteins in RAW264.7 cells treated with OE-*Pla2g7* and si-*Alcat1*, *n* = 3. **e** The protein expression of NLRP3, IL-1β, caspase-1, cleaved caspase-1, GSDMD, GSNMD^NT^, cGAS, and Sting in RAW264.7 cells treated with OE-*Pla2g7* and si-*Alcat1*. **f** Autophagosome acidification levels in macrophages treated with SiO_2_ and si-*Pla2g7*; scale bar = 10 μm. **g** Autophagosome acidification levels in macrophages treated with OE-*Pla2g7* and si-*Alcat1*; scale bar = 10 μm. **h** HE and Masson staining of lung tissue from Cre^lyz2^Pla2g7^fl/fl^ and control Pla2g7^fl/fl^ mice intratracheally treated with SiO_2_ and the autophagy inhibitor Baf A1 (scale bar = 20 μm). **i** The populations of SiglecF^lo^AMs in different experimental groups were analyzed via flow cytometry. **j** The protein expression of LC3I/II, TOM20, cytochrome C, and cathepsin B in macrophages treated with SiO_2_ and si-*Pla2g7*; *n* = 3. **k** Immunostaining of cathepsin B and LAMP1 in RAW264.7 cells treated with OE-*Pla2g7* or si-*Alcat1* (scale bar = 10 μm). **l** The protein expression of Col I, α-SMA, TOM20, and cytochrome C in the lung tissue of the mice in the different experimental groups. *n* = 3 per group. **m** The protein expression of Col I, α-SMA, TOM20, and cytochrome C in the lung tissue of the mice in the different experimental groups. *n* = 3 per group. Data are presented as the means ± SDs, *p < 0.05, **p < 0.01, ***p < 0.001, ^ns^ *p* ≥ 0.05

Mitophagy is essential for mitochondrial quality control. We further investigated the impact of Lp-PLA2 on mitophagy in SiO_2_-induced macrophages. Mitochondrial protein extraction revealed that SiO_2_ stimulation and OE-*Pla2g7* transfection increased the expression levels of the mitophagy initiation protein PTEN-induced putative kinase 1 (PINK1) and the autophagosome-forming protein LC3B in the mitochondria of macrophages. CL can modulate mitophagy in a manner dependent on or independent of the PTEN-induced putative kinase 1 (PINK1)/Parkin pathway. In our study, silencing *Pla2g7* or *Alcat1* further increased the expression levels of LC3B in mitochondria (Fig. 7a, b). However, silencing of *Alcat1* did not affect PINK1 expression in mitochondria (Fig. 7b), indicating that Lp-PLA2 and ALCAT1 function to inhibit mitophagy flux, whereas ALCAT1 regulates mitophagy independently of the PINK1 pathway. Whole-cell protein analysis revealed that intracellular protein ubiquitination and LC3II levels were increased in macrophages after SiO_2_ stimulation or OE-*Pla2g7* transfection. Similarly, the expression of the mitochondrial damage indicators translocase of the outer mitochondrial membrane20 (TOM20) and cytochrome C was upregulated. Inhibition of Lp-PLA2 or ALCAT1 can increase the expression level of LC3II, however, the levels of ubiquitination, TOM20 and cytochrome C were decreased (Fig. 7c, d and Supplementary Fig. S9a, b).

Furthermore, the silencing of *Alcat1* attenuated the promotion of pyroptosis proteins, including NOD-like receptor protein 3 (NLRP3), IL-1β, caspase 1, gasdermin D (GSDMD), and the activation of the pyroptosis regulatory pathway proteins cyclic GMP-AMP synthase (cGAS) and STING, in *Pla2g7*-overexpressing macrophages (Fig. 7e). Together, these findings are consistent with those of previous studies suggesting the existence of crosstalk between mitophagy and pyroptosis in macrophages [29, 30].

### The Lp-PLA2-ALCAT1-CL pathway induces incomplete mitophagy and pulmonary fibrosis through lysosomal damage

Autophagosomes fuse with lysosomes to form autolysosomes, which facilitate the degradation of damaged organelles. Impaired autolysosome formation leads to incomplete autophagy and sustained cellular stress. In this study, the *EGFP-mRFP-LC3* adenoviral vector was transfected to assess mitophagy integrity. This vector quenches the green fluorescence of EGFP and retains the red fluorescence of mRFP upon fusion with acidic lysosomes, indicating intact autophagy. Therefore, disruption of autophagosome‒lysosome fusion results in the retention of yellow fluorescence, indicating impaired autophagy. The findings revealed impaired autophagosome‒lysosome fusion following SiO_2_ induction and OE-*Pla2g7* transfection, as demonstrated by increased yellow fluorescence foci, which could be alleviated by *Pla2g7* and *Alcat1* silencing, resulting in red fluorescence foci (Fig. 7f, g).

Additionally, the expression level of the autophagic flux indicator LC3II was affected in Cre^Lyz2^Pla2g7^flox/flox^ mice (Supplementary Fig. S9d). With the use of the autophagosome‒lysosome fusion inhibitors bafilomycin A1 (Baf A1) and chloroquine, the results demonstrated that both Baf A1 and chloroquine attenuated the antifibrotic ability of Cre^Lyz2^Pla2g7^flox/flox^ mice (Fig. 7h and Supplementary Fig. S9c), resulting in the upregulation of TOM20, cytochrome C, Col I, and α-SMA expression in lung tissues (Fig. 7j, and Supplementary Fig. S9c). Furthermore, the ratio of SiglecF^lo^AMs in the lung tissues of SiO_2_-induced Cre^Lyz2^Pla2g7^flox/flox^ mice was lower than that in SiO_2_-induced Pla2g7^flox/flox^ mice; however, SiO_2_-induced Cre^Lyz2^Pla2g7^flox/flox^ mice treated with Baf A1 presented an increased proportion of SiglecF^lo^AMs (Fig. 7i).

Our results revealed that Ctsb, which encodes the lysosomal protein cathepsin B, was highly expressed in Pla2g7^high^ cells (Supplementary Figs. S5a and S9e). Cathepsin B degrades lysosomal contents when the lysosome is intact, however, when the lysosome is compromised, cathepsin B translocates to the cytoplasm, affecting autophagy flux [31] and TGF-β1 activation (Supplementary Fig. S9f) [32]. In this study, we found that the expression level of cathepsin B was significantly reduced in *Pla2g7* KO silicosis mice (Supplementary Fig. S9d). Subsequent detection of cathepsin B and the lysosomal-labeled protein Lamp1 revealed colocalization of Lamp1 and cathepsin B in OE-NC-transfected macrophages, whereas the Lamp1 level decreased in OE-*Pla2g7*-transfected macrophages, with cathepsin B dispersed throughout the cytoplasm, which could be reversed by silencing *Alcat1* (Fig. 7k). These results suggest that lysosomal damage in macrophages during silicosis is at least partially regulated by the Lp-PLA2-ALCAT1 pathway. Furthermore, *Pla2g7* and *Alcat1* silencing inhibited the increase in Cathepsin B and activation of the TGF-β-smad2/3 pathway in SiO_2_-stimulated and OE-*Pla2g7*-transfected RAW264.7 cells in vitro. (Fig. 7l, m). These results indicate that the ALCAT1-Lp-PLA2 pathway compromises mitophagy integrity, thereby disrupting autophagy flux by regulating lysosomal damage.

### Lp-PLA2-specific oral inhibitor darapladib protected mice from silica exposure-induced pulmonary fibrosis

**Fig. 8:**
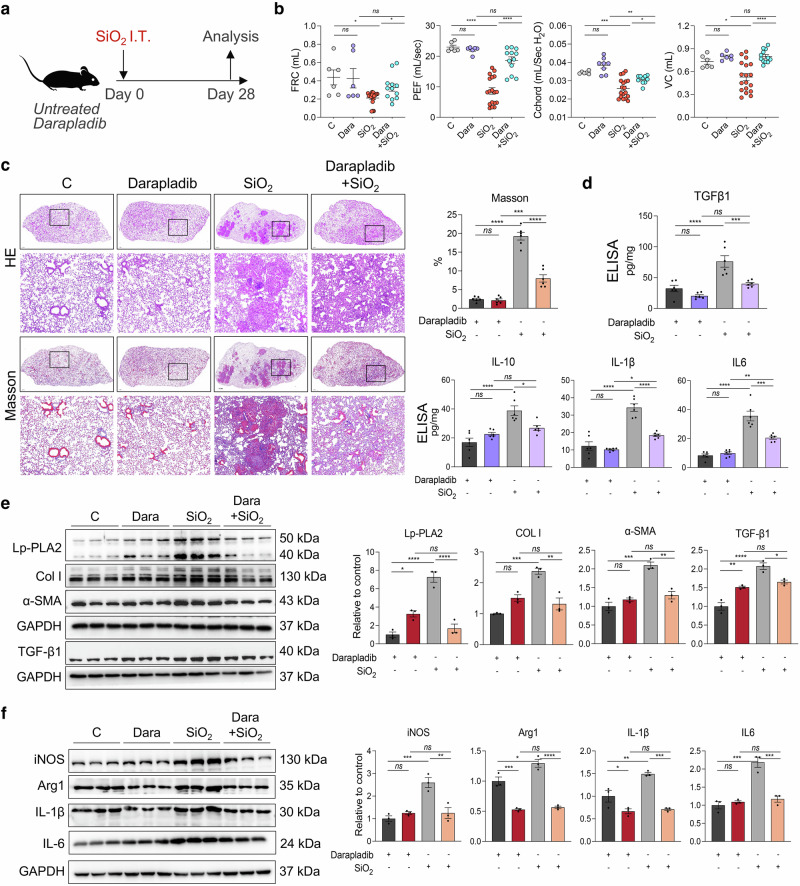
Antifibrotic effect of the Lp-PLA2 specific inhibitor darapladib on silicotic mice. **a** Schematic diagram of silicotic mice treated with darapladib. **b** Lung function measurements of FRC, PEF, Cchord, and VC in mice from different experimental groups. *n* = 9 per group. **c** Lung tissue morphology and collagen deposition detected by HE and Masson staining; scale bar = 1 mm; zoom bar = 50 μm. **d** The protein expression levels of iNOS, Arg 1, IL-1β, and IL-6 in mouse lung tissues were examined via ELISA. *n* = 6 per group. **e** Western blot analysis was performed to detect the expression of Lp-PLA2, α-SMA and Col I in mouse lung tissues, *n* = 3. **f** Western blot analysis was performed to detect the expression of iNOS, Arg 1, IL-1β, and IL-6 in mouse lung tissues. *n* = 3 per group. The data are presented as the means ± SDs; *p < 0.05, **p < 0.01, ***p < 0.001, ^ns^ *p* ≥ 0.05

We conducted a randomized controlled trial to assess the efficacy of darapladib, a specific oral inhibitor of Lp-PLA2, in treating silica-induced pulmonary fibrosis in mice (Fig. 8a). Our findings revealed that, compared with control mice, darapladib-treated mice did not exhibit significant differences in lung function, pulmonary tissue morphology, or collagen deposition. However, mice exposed to SiO_2_ and subsequently treated with darapladib presented significant improvements in lung function and reduced severity of pulmonary fibrosis compared with those treated with a solvent control (Fig. 8b, c). Western blotting analysis revealed that the expression levels of fibrotic proteins (Col I, α-SMA, and TGF-β1) and inflammatory proteins (iNOS, Arg1, ΙL-1β, and ΙL-6) in the lung tissues of the mice in the SiO_2_+darapladib group were significantly lower than those in the SiO_2_ group (Fig. 8e, f). Similar results were also obtained by ELISA (Fig. 8d). Moreover, lipidomic results further revealed that darapladib impedes the acylation and unsaturation of CL (Supplementary Figure 10).

## Discussion

The lung functions as the primary interface between the external and internal environments and is consistently exposed to numerous inorganic and organic particles in the air. Pulmonary tissue has developed specialized immune mechanisms to defend against external threats, and lung macrophages are essential for coordinating immune responses and preserving tissue homeostasis amid environmental challenges [9, 11, 12]. The pathophysiology of silicosis-induced lung injury involves complex immune dysregulation mechanisms, as demonstrated in our study through the integration of scRNA-seq, ST-seq, and lipidomic analyses. We revealed a deviation in the differentiation pathway of MoMacs after silica-induced pulmonary injury, resulting in the emergence of a distinct subset termed Spp1^hi^Macs [33], which has been identified as a significant factor in the progression of fibrosis observed in silicosis. Spp1^hi^Macs exhibit overlapping characteristic genes with a fibrosis-inducing alveolar macrophage cluster identified in asbestos-induced pulmonary fibrosis mouse models and patients with idiopathic pulmonary fibrosis [15]. A previous study reported that immaturely differentiated SiglecF^lo^AMs induce pulmonary fibrosis [15]. Consistent with these findings, our study revealed a progressive increase in the percentage of SiglecF^lo^AMs within silicosis-afflicted lungs, a trend that corresponds with Spp1^hi^Macs. Moreover, Spp1^hi^Macs characteristic gene expression in SiglecF^lo^AMs was upregulated compared to that in mature-differentiated SiglecF^hi^AMs, indicating that Spp1^hi^Macs resemble a transitional immature state. MoMacs retain partial inflammation-related phenotypic traits during injury and lung inflammation, increasing their reactivity to subsequent stimuli. However, the cessation of inflammatory signals under stable conditions is essential for MoMacs to develop transcriptional traits similar to those of tissue-resident macrophages [12]. These findings provide implications for the different functions of Spp1^hi^Macs and TRAMs in inflammation, fibrotic repair, and collagen synthesis observed in this study. Spp1^hi^Macs exhibit strong M1 and M2 polarization, which may be attributed to dysregulation and incomplete differentiation of Spp1^hi^Macs during proinflammatory and fibrotic repair and contributes significantly to silicosis.

Significantly increased lipid peroxidation is detected in the peripheral blood and exhaled gas of patients with pneumoconiosis, and the expression level of Lp-PLA2 can be induced by lipid peroxidation [18, 34]. This may account for the persistent high expression of Lp-PLA2 in monocytes and macrophages in silicosis. Pla2g7^high^ macrophages have been reported to facilitate fibroblast-to-myofibroblast transition through activation of the phosphatidylcholine-lysophospholipid metabolic pathway [35]. PLA2s function as regulatory checkpoints in lipid mediator metabolism. The primary role of Lp-PLA2 involves hydrolyzing ester bonds at the sn-2 position of unsaturated phospholipids, producing lipid mediators, including arachidonic acid, linoleic acid, prostaglandins (PGD), leukotrienes, thromboxane (TXA), lipoxins, and platelet-activating factor, which regulate inflammation [20, 26, 27]. Our previous studies demonstrated that PGD2 and TXA2 facilitate the onset and progression of silicosis [21]. Moreover, Lp-PLA2 modulates immune cell function by influencing membrane phospholipid fatty acid rearrangement through its unique affinity for these substrates [19, 20, 36]. We found that *Pla2g7* overexpression or silencing influences M1 and M2 polarization of SiO_2_-induced macrophages, thereby regulating their proinflammatory and profibrotic functions. Specific deletion of *Pla2g7* in macrophages effectively inhibited pulmonary fibrosis in silicosis model mice and reduced the proportion of  SiglecF^lo^AMs. Thus, Lp-PLA2-associated lipid mediator metabolic pathways constitute a core link between lipid metabolism, inflammation, and monocyte‒macrophage phenotypes during silicosis progression.

Our findings revealed a significant increase in the acylation and unsaturation levels of mitochondrial membrane-specific lipid CL substrates in AMs from silicotic mice. The various acyl chain components of the CL can affect the formation of respiratory chain supercomplexes, confer resistance to ROS damage, and preserve mitochondrial homeostasis [37, 38]. In response to injury stimuli, the CL undergoes acylation, desaturation, and oxidation facilitated by ALCAT1 catalysis, resulting in its translocation from the inner to the outer mitochondrial membrane and subsequent mitochondrial damage-associated inflammatory responses [28]. The quantity, status, and metabolic form of mitochondria affect silicosis fibrosis development [30] and regulate the survival and differentiation of monocytes and macrophages [39]. We found that damaged mitochondria accumulate in MoMacs affected by silicosis and that the silencing of *Pla2g7* mitigated SiO_2_-induced changes in ALCAT1 levels. Moreover, SiO_2_ stimulation or *Pla2g7* overexpression induced a decrease in the mitochondrial membrane potential and an increase in mtROS, both of which could be reversed by silencing *Pla2g7* or *Alcat1*. CL can activate mitochondrial fission and fusion, thereby improving mitochondrial tolerance. Consistently, our study revealed that Lp-PLA2 promotes mitochondrial fission and inhibits mitochondrial fusion through ALCAT1. Taken together, these findings suggest that the activation of the Lp-PLA2-ALCAT1-CL pathway is a significant factor contributing to mitochondrial damage in MoMacs during silicosis.

This study presents new evidence that the Lp-PLA2-ALCAT1-CL pathway inhibits mitophagy. Studies have shown that CL can trigger mitochondrial autophagy, either dependent on or independent of the PINK1/Parkin pathway, to prevent the detrimental effects of damaged mitochondria on cells [40, 41]. We found that silencing *Alcat1* did not affect PINK1 expression in the mitochondria of *Pla2g7*-overexpressing macrophages, indicating that CL-related mitochondrial autophagy pathways are influenced by Lp-PLA2 levels in silicosis, via PINK1/Parkin-dependent and PINK1/Parkin-independent pathways.

Iriondo et al. compared the binding efficiency of the LC3 subfamily to CL during CL-associated mitochondrial autophagy and reported that CL oxidation levels inhibit the binding of LC3B to CL, whereas LC3A is unaffected by CL oxidation levels [42]. Here, the regulation of mtROS levels by Lp-PLA2 clarifies the oxidation mechanism of CL in silicosis, with the increased affinity of Lp-PLA2 for oxidized phospholipids [43], exacerbating the CL-mediated mitochondrial autophagy process. However, in macrophages subjected to silicosis with high mtROS levels, the unsaturation and oxidation of CL hinder its recruitment of LC3B, affecting autophagic flux and the integrity of mitophagy. Accordingly, we found that silencing *Alcat1* effectively inhibited the Lp-PLA2 overexpression-induced increase in ubiquitination, reduction in autophagic flux, and acidification defects, thereby preventing incomplete mitophagy. These findings suggest that the status of the CL (including unsaturation, acylation, and oxidation) profoundly impacts both the initiation and integrity of mitophagy.

Additionally, in *Pla2g7*-overexpressing macrophages, the level of the lysosomal marker LAMP1 was also reduced, suggesting that lysosomal damage leading to impaired autolysosome formation may play a significant role in incomplete mitophagy. Lysosomal compromise results in the translocation of cathepsin B to the cytoplasm, affecting autophagic flux [31] and TGF-β1 activation. Our in vivo studies demonstrated that, compared with Pla2g7^fl/fl^ silicosis mice, Cre^Lyz2^Pla2g7^fl/fl^ silicosis mice presented lower expression levels of cathepsin B. Furthermore, Cre^Lyz2^Pla2g7^fl/fl^ silicosis mice treated with an autophagy inhibitor to disrupt autophagic lysosome processes exhibited exacerbated lung fibrosis, mitochondrial damage, and a restored proportion of SiglecF^lo^AMs. As a result, Lp-PLA2-ALCAT1-CL diminishes the affinity between LC3B and the CL or induces lysosome damage, thereby compromising autophagy integrity, which may significantly contribute to the differentiation disorders of MoMacs.

A significant aspect of our work was the assessment of the therapeutic efficacy of Lp-PLA2 inhibition in silicosis. Although darapladib, the most advanced Lp-PLA2 inhibitor, failed to meet the primary endpoint in extensive phase III trials involving patients with atherosclerosis and stable coronary heart disease receiving standard medical treatment [44, 45], the study of Lp-PLA2 remains ongoing [27]. Recent clinical and preclinical studies have reported that darapladib confers clinical benefits in patients with diabetic macular edema, Alzheimer’s disease, and drug-tolerant cancer [46] because of its regulation of lipid mediator metabolism, which reduces microvascular damage and necrotic core volume expansion [45]. Considering that silicosis is associated with pulmonary capillary depletion [9, 47] and that monocyte necrosis is essential for monocyte-to-macrophage differentiation and maturation [15], assessing the potential clinical application value of darapladib in silicosis is imperative.

In conclusion, this study demonstrated the intricate interplay between lipid metabolism and mitochondrial dysfunction in the pathogenesis of the silicosis-induced macrophage profibrotic phenotype, and provides a framework for investigating therapeutic interventions targeting macrophage-mediated inflammation and fibrosis in lung diseases.

## Materials and methods

### Human samples

Lung tissue samples from patients diagnosed with silicosis were obtained from lung transplant surgeries conducted at the China-Japan Friendship Hospital in Beijing, China. Lung tissue samples from 3 patients were used for immunostaining. These patients had no history of other pulmonary diseases. All participants provided written informed consent prior to inclusion in the study. Ethical approval for this study was obtained from the Clinical Research Ethics Committee of the China-Japan Friendship Hospital (Beijing, China) (2022--KY-031), and all procedures were conducted in accordance with the ethical principles outlined in the 1975 Helsinki Declaration.

### Animals

All animal experiments were conducted in accordance with international guidelines for animal experimentation. The research protocol was approved by The Medical College of Beijing Municipal Commission of Animal Protection and Utilization (Beijing, China) and the Animal Studies Committee of the China-Japan Friendship Hospital (license no. zryhyy21-21-04-01). Female C57BL/6J mice were purchased from Beijing Vital River Laboratory Animal Technology Co., Ltd. (Beijing, China), and Cre^Lyz2^Pla2g7^fl/fl^ mice were constructed from Cyagen Biosciences (Suzhou, China) on a C57BL/6J background.

Eight-week-old male mice were selected for the experimental study. The silicosis model was induced via single intratracheal instillation of silica particles via a previously described protocol. Briefly, SiO_2_ (5 μm particles, s5631, Sigma, USA) particles were ground for 2 h and then heat-treated at 200 °C for 2 h to ensure sterilization. SiO_2_ was subsequently dissolved in phosphate-buffered saline (PBS) to prepare a suspension with a concentration of 300 mg/ml. The mice were anesthetized via isoflurane, and the silica-exposed group (*n* = 9) received an intratracheal instillation of 20 μl of silica suspension, whereas the control group (*n* = 9) received 20 μl of PBS via the same route. For darapladib (*n* = 9), bafilomycin A1 (Baf A1) (*n* = 9) or chloroquine treatment (*n* = 9), the mice received darapladib (25 mg·kg^−1·^d^−1^, reconstituted in saline) by gavage once daily, Baf A1 (1 mg·kg^−1^·d^−1^, reconstituted in saline) by intrabitoneal injection once daily, and chloroquine (10 mg·kg^−1^·d^−1^, reconstituted in saline) by intrabitoneal injection once daily. The corresponding control group was treated with saline. At designated time points postexposure, the mice were euthanized, and lung tissue samples were collected for histological, biochemical, and molecular analyses to assess the development and progression of silicosis.

### Cell culture

The mouse monocyte macrophage line RAW264.7 and the mouse alveolar epithelial cell line MLE-12 were purchased from Wuhan Pricella Biotechnology Co., Ltd. (Wuhan, China). Two types of cells were cultured in their respective specialized culture media in a humidified atmosphere at 37 °C and 5% CO_2_. Primary fibroblasts were extracted as previously described [48], and the third generation was used for this study. BMDMs were obtained as previously reported [49]. Upon lysis of the bone marrow red blood cells obtained from the mice, the cells were resuspended in RPMI 1640 medium supplemented with 10% fetal bovine serum (FBS), penicillin/streptomycin, and 30 ng/ml macrophage colony-stimulating factor (M-CSF). The culture medium was changed every two days over a period of seven days. Where indicated, the cells were treated with silica (50 μg/cm^2^) in serum-free media for 24 h. For transfections, the cells were cultured in antibiotic-free media.

### Lipidomics

Sample preparation and metabolic profiling were performed with standard procedures in cooperation with Applied Protein Technology (Beijing, China). Briefly, lipids were extracted according to the MTBE method. LC‒MS/MS method for lipid analysis. Polled quality control (QC) samples were set in the sample queue to evaluate the stability and repeatability of the system. MultiQuant or Analyst was used for quantitative data processing. Metabolites in the QCs whose coefficient of variation (CV) was less than 30% were denoted as reproducible measurements.

### Micro-CT imaging

The mice were anesthetized via isoflurane, and high-resolution CT images of mouse lungs were obtained via the NEMO-II NMC-200 machine (Pingsheng, Jiangsu, China) in accordance with the manufacturer’s protocol as previously reported. The acquired images were processed via AVATAR 1.5.0 software to generate three-dimensional representations.

### Pulmonary function measurement

The FinePointe PFT system (Buxco Research Systems, Wilmington, NC, USA) was used to gauge pulmonary functions in accordance with the manufacturer’s protocol. After the mice were subjected to intraperitoneal anesthesia, tracheostomy and tracheal intubation were performed. Under mechanical respiration, vital capacity (VC), enlarged peak expiratory flow (PEF), functional residual capacity (FRC), and chord compliance (Cchord) are measured via pressure evolution manipulation.

### Fluorescence-activated cell sorting (FASC) and flow cytometry (FC)

The mice were euthanized, and the lungs were removed. The lung tissues were immersed in saline containing 2 mg·mL^−1^ collagenase D (Roche, Indianapolis, IN, USA) and 0.2 mg·mL^−1^ DNase I (Roche). The lung tissue was cut into approximately 1 mm^3^ pieces and incubated at 37 °C for 60 min with gentle stirring. A 30 G needle was used to scratch the tissue clumps. The single-cell suspension was filtered through a 40 μm nylon cell strainer to obtain a single-cell suspension. The cells were incubated with anti-mouse CD45 microbeads (Miltenyi Biotech), and CD45+ cells were collected via a MultiMACS Cell24 Separator (Miltenyi Biotech) according to the manufacturer’s protocol. The cells were stained with Zombie Aqua™ fixable viability dye (eBioscience, USA), incubated with TruStain FcX™ PLUS (BioLegend, USA) and stained with the mixture of fluorochrome-conjugated antibodies listed in the supplementary materials (Supplementary Table S1) in accordance with the concentrations recommended in the instructions. Data were acquired via a BD FACSCanto™ instrument and BD FACSDiva software (BD Biosciences, USA) or Backman Coulter Cytoflex™ instrument and Cytoflex. Software (Backman Coulter Life Sciences, Germany) was used. The cell population was identified via a sequential gating strategy (Supplementary Fig. S4b). Data analysis was performed via FlowJo software.

### Mitochondrial membrane potential (MMP)

MitoTracker Deep Red 633 (Beyotime Biotechnology, Shanghai, China) was used to measure the MMP. RAW264.7 cells were stimulated according to the study protocol and cocultured with 100 nM MitoTracker Deep Red 633 for 30 min. Then, the cells were washed twice with PBS. Single-cell suspensions were examined via flow cytometry using a Beckman Coulter Cytoflex™ instrument and CytoFlex software (Beckman Coulter Lifesciences, Germany). Stained mitochondria were analyzed via FlowJo software.

### Mitochondrial ROS (mtROS)

The presence of mtROS was determined via the use of a MitoSOX indicator (MedChemExpress, Shanghai, China). In brief, after stimulation with stuty policotol, RAW264.7 cells were incubated with 5 µM MitoSOX for 20 min and washed twice with PBS. The level of mitochondrial ROS was measured via flow cytometry via the same equipment used for the MMP measurement.

### Autophagic flux

Autophagic flux was assessed via mRFP-EGFP-LC3B adenoviruses obtained from Hanbio (Shanghai, China) according to the manufacturer’s protocols. RAW264.7 cells were transfected with mRFP-EGFP-LC3B at a MOL of 250. The appearance of LC3-positive autophagosomes and autolysosomes was examined via fluorescence confocal microscopy (Nikon, Tochigi, Japan) and NIS-Element Viewer software (Nikon, Tochigi, Japan).

### Enzyme-linked immunosorbent assay (ELISA)

ELISAs were performed as previously described [50]. The protein levels of TGFβ1, IL-10, IL-1β and IL-6 in mouse lung tissue were detected via ELISA kits according to the instructions of the kits. See Supplementary Table S2 for specific kit information.

### Statistical analysis

SPSS 22.0 statistical software and GraphPad Prism software were used for data analysis. Data from at least three independent experiments are expressed as the mean ± SD. For comparisons of differences between groups, one-way ANOVA or an independent-sample t test was conducted. The significance of the lipidomic data was determined via an unpaired Student’s *t* test. *p*  <  0.05 was considered statistically significant.

## Supplementary information

Revised Supplementary Materials

## Data Availability

The data supporting the findings of this study are available from the corresponding authors upon reasonable request.
