# Supplementary material for: Targeting Lp-PLA2 inhibits profibrotic monocyte-derived macrophages in silicosis through restoring cardiolipin-mediated mitophagy
Source: Cell Mol Immunol. 2025 May 19;22(7):776–90. doi: 10.1038/s41423-025-01288-5 (PMC12206922; doi:10.1038/s41423-025-01288-5)
Supplement: Supplementary file 1 — Revised Supplementary Materials [file 41423_2025_1288_MOESM1_ESM.docx]

**Supplementary Materials for**

**Targeting Lp-PLA2 inhibits pro-fibrotic monocyte-derived macrophages in silicosis through restored cardiolipin-mediated mitophagy**

Shifeng Li^a^, Hong Xu^b^, Shupeng Liu^c^, Jinkun Hou^a^, Yueyin Han^a^, Chen Li^a,d^, Yupeng Li^a,e^, Gaigai Zheng^c^, Zhongqiu Wei^f^, Fang Yang^c^, Shuwei Gao^a^, Shiyao Wang ^a^, Jing Geng^a^*, Huaping Dai^a^*, Chen Wang^a^*

^a^ National Center for Respiratory Medicine, State Key Laboratory of Respiratory Health and Multimorbidity, National Clinical Research Center for Respiratory Diseases, Institute of Respiratory Medicine, Chinese Academy of Medical Sciences, Peking Union Medical College, Immune Dysfunction and Pulmonary Fibrosis Joint Laboratory for Clinical Medicine, Capital Medical University, Department of Pulmonary and Critical Care Medicine, China-Japan Friendship Hospital, Beijing, China.

^b^ Health Science Center, Hebei Key Laboratory of Organ Fibrosis, North China University of Science and Technology, Tangshan, Hebei, China.

^c^ School of Public Health, Hebei Key Laboratory of Organ Fibrosis, North China University of Science and Technology, Tangshan, Hebei, China.

^d^ Capital Medical University, Beijing, China.

^e^ Second Affiliated Hospital of Harbin Medical University, Respiratory and Critical Care Medicine, Harbin, Heilongjiang, China.

^f^ Basic Medical College, Hebei Key Laboratory of Organ Fibrosis, North China University of Science and Technology, Tangshan, Hebei, China.

* **Corresponding author**

Department of Pulmonary and Critical Care Medicine, China-Japan Friendship Hospital, No. 2 East Yinghua Road, Chaoyang District, Beijing, 100029, China.

E-mail addresses: [jing_geng@foxmail.com](mailto:jing_geng@foxmail.com) (Jing Geng) [daihuaping@ccmu.edu.cn](mailto:daihuaping@ccmu.edu.cn) (Huaping Dai); [cyh-birm@263.net](mailto:cyh-birm@263.net) (Chen Wang).

**This word file includes:**

Materials and Methods

Supplementary Text

Supplementary Figures S1 to S10

Tables S1 to S2

**Supplementary Materials and Methods**

**Single-cell RNA sequencing**

Initial data processing was performed using the Cell Ranger version 4.0.0 pipeline (<https://www.10xgenomics.com/>), reads were alignment to the mm10 version of the mouse genome. Follow-up analysis was performed using the R package of Seurat version 4.3.0 and was based primarily on the official tutorial. Cells with nFeature_RNA > 200 and percent.mt < 15 and genes expressed in less than 3 cells were filtered. The cells are then subjected to principal component analysis (PCA), and Uniform Manifold Approximation and Projection (UMAP) to find cell clusters^48^. Batch effects among four samples were alleviated with Harmony^49^. Difference analysis and cell annotation was performed to obtain the proportional of cell clusters and subclusters of silicosis lung. Analysis of the pseudotime in single cells was performed using the Monocle R package to obtain the trajectory of cell differentiation and the key genes and mechanisms involved in cell differentiation.

**Spatial RNA sequencing**

The lung tissue section was adhered to the surface of a glass slide and stained with HE. The lung tissue section was then permeabilized and prepared into an information library containing barcodes. Subsequently, the cells were clustered, and the locations of different niches in different original spatial positions were determined using R software and Seurat package of version 4.3.0.

**Lentivirus transfection**

Lentivirus of Pla2g7 and Alcat1 silence, Pla2g7 overexpression, and all negative controls were obtained from Genechem (Shanghai, China). The RAW264.7 cells were transfected with the above lentivirus according to the manufacturer’s instructions. The cells were screened with puromycin for 7 days.

**Histopathological Examination**

Lung tissue samples were fixed in formalin and embedded in paraffin blocks. Sections of 5 µm thickness were prepared and stained with hematoxylin and eosin (H&E) (Solarbio Lifescience) for routine histopathological examination. Masson (Solarbio Lifescience) staining were employed to assess fibrosis. Histopathological analysis was performed by experienced pathologists blinded to the clinical data.

**Immunofluorescence**

Lung tissues were deparaffinized to hydrate, followed by antigen repairment. Slides were incubated with the indicated primary antibodies overnight at 4 °C. Subsequently, the slides were incubated with secondary anti-rabbit or anti-mouse IgG. Stained slides were captured with fluorescence confocal microscopy (Nikon, Tochigi, Japan) and the NIS-Element Viewer software (Nikon, Tochigi, Japan) or scanned with a Pannoramic Scan 250 Flash or MIDI system, and images obtained by using Pannoramic Viewer 1.15.2 (3DHistech, Budapest, Hungary). Antibodies and the related reagents were listed in the Supplementary Table S1.

**Western blot**

Protein lysates of cells or tissue samples were extracted with cell lysis buffer (Solarbio Lifescience, Beijing, China) containing protease inhibitor (Solarbio Lifescience, Beijing, China). 10 μg volumes of protein samples were separated by 10% SDS-PAGE and electro-transferred to PVDF membranes (IPVH00010, Merck Millipore, Billerica, MA, USA). The immunoblots were probed with the indicated antibodies. Proteins bands were visualized with NcmECL High Enhanced Chemiluminescent (NCM Biotech, Shanghai, China). Quantification of bands in Western blotting were performed using Image-Pro Plus software (Media Cybernetics, Rockville, Maryland, USA). The results were normalized with a loading control and were expressed as fold changes relative to specific bands in the control group. Western blotting was repeated at least three times. Antibodies and the related reagents were listed in the Supplementary Table S1.

**Quantitative polymerase chain reaction (****qPCR)**

The qPCR was performed as previously described. Total RNA was extracted from cells or tissue samples with TRIzol reagent (Invitrogen, Carlsbad, CA, USA). Reverse transcription (K1622, Thermo Scientific, USA or ZR102, ZOMANBIO, China) was performed according to the manufacture’s procedures. The mRNA levels were quantitated by using 2× SYBR qPCR Mix (ZF102, ZOMANBIO, China) system, and normalized to gapdh expression. Additional information about all the primers in this study were listed in the Supplementary Table S2.

**Transmission electron microscopy**

Cells were collected from different treatments and fixed with 2.5% glutaraldehyde and then fixed with 1% osmic acid. After alcohol gradient dehydration, the samples were resin embedded. Dropped 2% phosphotungstic acid on the copper grid to stain for 1–2 min, used filter paper to absorb excess liquid, and dry at room temperature. The copper grids are observed under TEM and taken images.

**Supplementary Text**

**Supplementary Figure S1 Cellular composition in mouse lung delineated by scRNA-seq analysis.**

(a) UMAP plots showing the cell colored by samples. (b) Bar plot demonstrating the cellular composition in cells from silicosis and control mouse. (c) Feature plots demonstrating the expression of cluster-specific genes: Macrophages are characterized by expression of *Lyz2* and *Cd68*, *Clec4a1* and *Cx3cr1* as IM markers, *Csf1* as a marker of monocyte, DCs are characterised by expression of *Ccl2*, *Gzma* as NK cell marker, *Retnlg* as Neutrophil marker, *Cyp11a1* as Mast cell marker, *Cd3d* as T cell marker, *Ly6d* as B cell marker, *Pecam1* and *Sftpc* as epithelial cell markers, *Col3a1* as fibroblast marker.

**Supplementary Figure S2 The Dot plots demonstrate function of macrophage subclusters.**

The dot plots show the GO enrichement of characteristic genes in macrophage subclusters.

**Supplementary Figure S3 Tissue niche composition in silicosis lung delineated by ST-seq analysis.**

(a) t-SNE plots showing seven niches in mild, medium, and severe silicosis lung samples delineated by ST-seq. (b) Distribution of the seven niches in the four samples. (c) The barcode dot plot shows the tissue niche of silicosis lungs. (d) The expression of characteristic genes in each niche.

**Supplementary Figure S4 Gating strategy used to FC and FACS-sort populations.**

(a) H&E and Masson staining of mouse lung tissue of control (day 0), inflammation (day 7) and fibrosis (day 28). (b) Gating strategy used to FC and FACS-sort populations of IMs, AMs, and SiglecF^hi^AMs, SiglecF^lo^AMs during SiO_2_-induced lung fibrosis.

**Supplementary Figure S5 Expression of key gene Spp1 in Pla2g7^high^ macrophages and lung niches, and Enrichment analysis of KEGG pathway in silicosis niche.**

(a) Volcano diagram shows differential genes between Pla2g7^high^ and Pla2g7^low^ macrophages. (b) Expression of *Spp1* in different lung tissue niches. (c) Enrichment analysis of KEGG pathways of top 50 genes in ST-seq fibrotic niche.

**Supplementary Figure S6 Generation and validation of Cre^Lyz2^Pla2g7^flox/flox^ mice.**

(a) Experimental scheme for generating the Cre^Lyz2^Pla2g7^flox/flox^ mice. (b) The expression of the *Cre* and *Pla2g7* genes was verified by PCR. WT and water were used as negative control and blank control, respectively.

**Supplementary Figure. S7** **Effect of Pla2g7 on M1 and M2 polarization in SiO_2_-induced RAW264.7.**

(a) The protein expression of Lp-PLA2, caspase1, IL-1β, IL-10, pStat6 and Stat6 in RAW264.7 cells following OE-*Pla2g7* transfection and SiO_2_ stimulation detected by western blot, n = 3. (b) The expression of IL-1β and caspase-1 were detected by immunofluorescence in RAW 264.7 cells induced by SiO_2_+si-Pla2g7, scale bar = 10μm. (c) Co-localization of IL-1β and IL-10 in RAW264.7 cells following OE-Pla2g7 transfection and SiO_2_ stimulation detected by immunofluorescence, scale bar = 10 μm. (d) Co-localization of caspase1 and TNF-α in RAW264.7 following OE-Pla2g7 transfection and SiO_2_ stimulation detected by immunofluorescence, scale bar = 10 μm. (e) Expression of α-SMA in alveolar epithelial cells (MLE12) and primary fibroblasts co-culture with macrophages following OE-Pla2g7 transfection and SiO_2_ stimulation, scale bar = 20 μm. Data are presented as the mean ± SD, **p* < 0.05, ***p* < 0.01, ****p* < 0.001, ^ns^ *p* ≥ 0.05.

**Supplementary Figure. S8 Metabolic pathway analysis macrophages in bronchial alveolar lavage fluid (BALF) of pneumoconiosis patients (GSE174725) and mouse lung tissue.**

(a) Lung imaging of pneumoconiosis patients (CT). (b) UMAP plots of cell clusters in BALF of pneumoconiosis patients. (c) UMAP plots of macrophage subclusters in BALF of pneumoconiosis patients. (d) Enrichment map of differential metabolic pathways between cluster C1(increased in silicosis lung) and C0 (decreased in silicosis lung). (e) PCA analysis of lipidomics of AMs sorted from lung tissue of control and silicosis mice. (f) Bubble diagram of lipid subclass. (g) The level of mitoSOX in SiO_2_-induced macrophage treated with SS-31, scale bar = 20 μm.

**Supplementary Figure S9 Regulation of the Lp-PLA2-ALCAT1-CL pathway on mitophagy and cathepsin B-TGF-β1 signaling in RAW264.7 cells and mouse lung tissue.**

(a) The protein expression of LC3 I/II, TOM20, cytochrome C in RAW264.7 cells treated with SiO_2_ + si-Pla2g7, (b) and OE-Pla2g7 + si-Alcat1. (c) The protein expression of Col I, α-SMA, TOM20, and cytochrome C in lung tissue of mice treated with SiO_2_ intratracheal administration and autophagy inhibitor chloroquine, scale bar = 20 μm. (d) The expression of cathepsin B and LC3 I/II in lung tissue of four groups (Pla2g7^flox/flox^, Cre^Lyz2^Pla2g7^flox/flox^, Pla2g7^flox/flox^ + SiO_2_, Cre^Lyz2^Pla2g7^flox/flox^ + SiO_2_) examined by western blot. (e) Co-localization of Lp-PLA2 and cathepsin B in lung tissues of silicosis patients detected by immunofluorescence, scale bar = 50 μm. (f) The expression of TGF-β1 and cathepsin B was detected by immunofluorescence in lung tissue of silicosis mouse, scale bar = 100μm. The data are reported as mean ± SE (n=3 mice per group). Data are presented as the mean ± SD, **p* < 0.05, ***p* < 0.01, ****p* < 0.001, ^ns^ *p* ≥ 0.05.

**Supplementary Figure S10 Lung tissue macrophages of silicosis mice treated with darapladib were analyzed by lipidomics.**

(a) PCA analysis of lipidomics of AMs sorted from lung tissue of control, silicosis and darapladib-treated silicosis mice. (b) Heat map of CL acylation level. (c) Volcano plots showing the differences in lipids of lung macrophages between SiO_2_ vs. control group and darapladib vs. SiO_2_ group. (d) The saturation degree of different carbon units of CL.


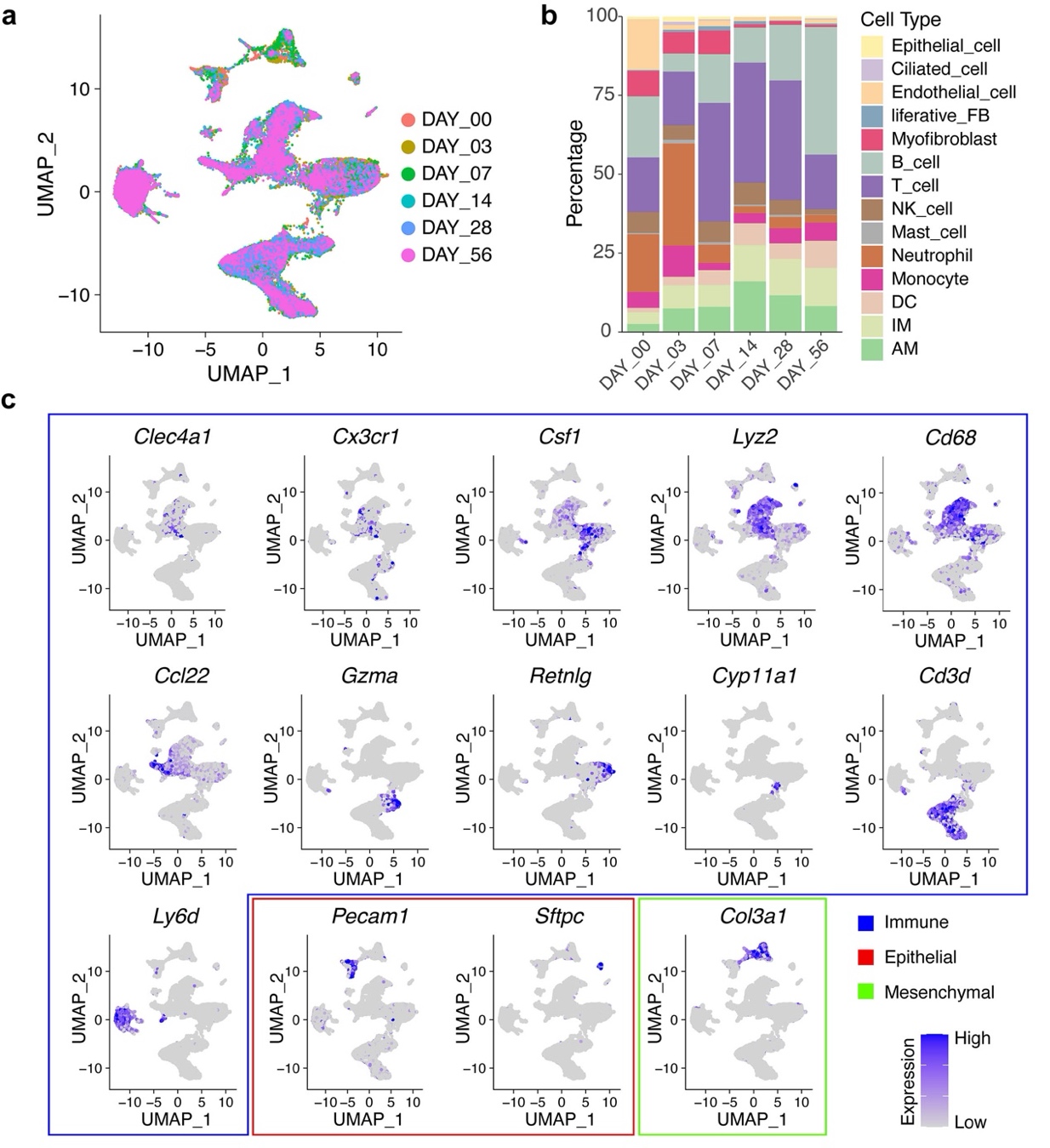


**Figure. S1** **Cellular composition in mouse lung delineated by scRNA-seq analysis**

**
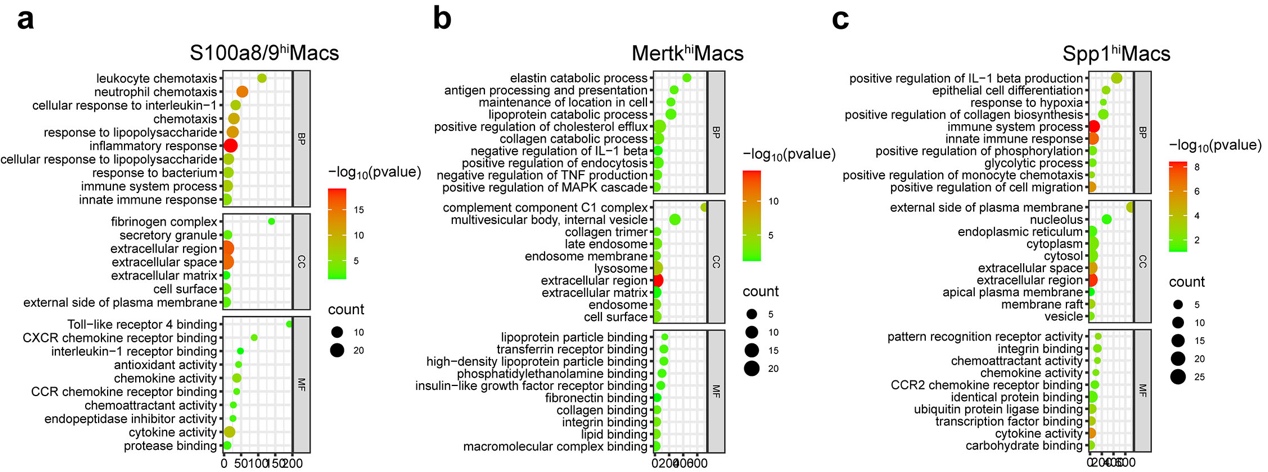
**

**Figure. S2** **The Dot plots demonstrate function of macrophage subcluster**

**
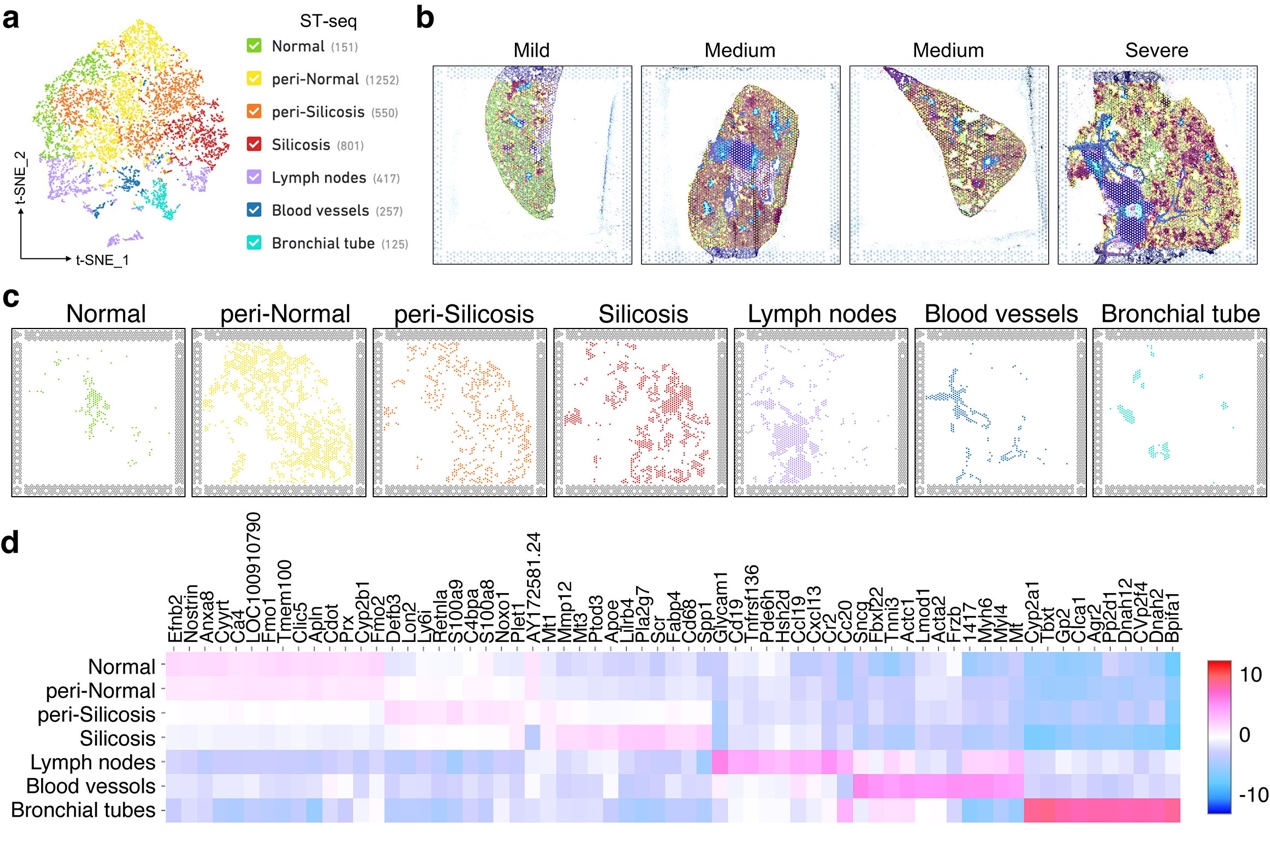
**

**Figure. S3 Tissue niche composition in silicosis mouse lung delineated by ST-seq analysis.**

**
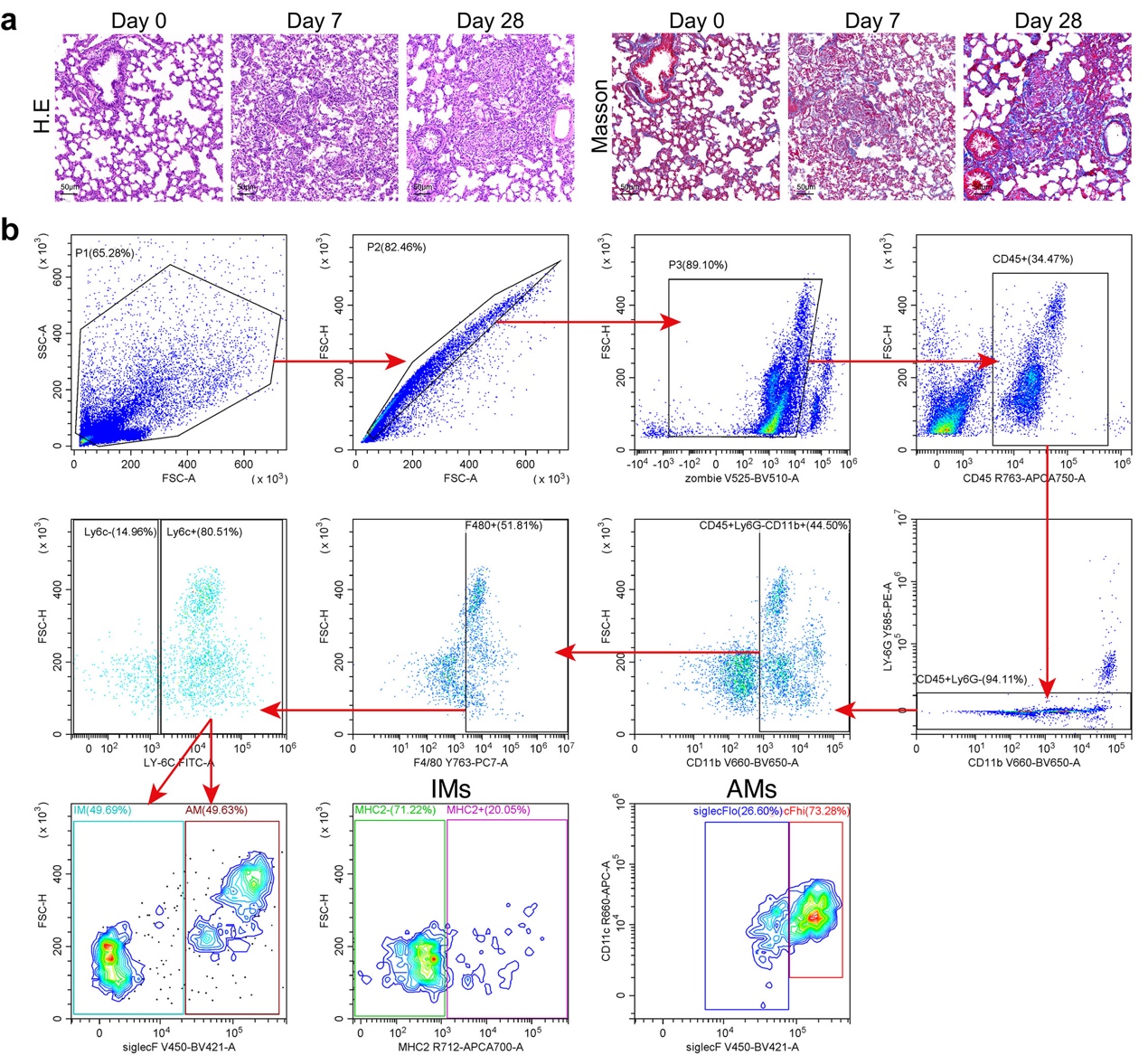
**

**Figure. S4 Gating strategy used to FC and FACS-sort populations.**


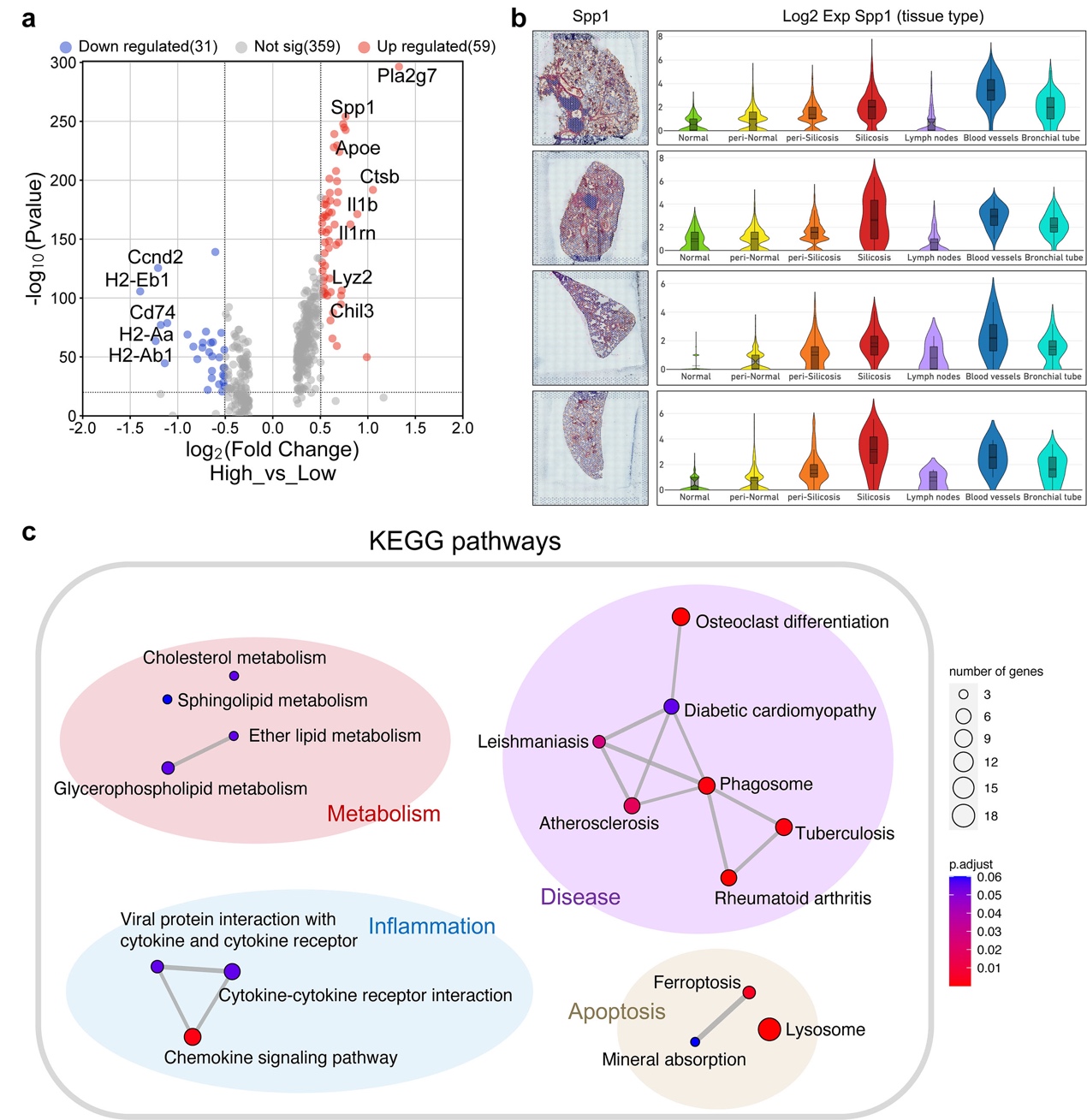


**Figure. S5 Expression of key gene Spp1 in Pla2g7^high^ macrophages and lung niches, and Enrichment analysis of KEGG pathway in silicosis niche.**

**
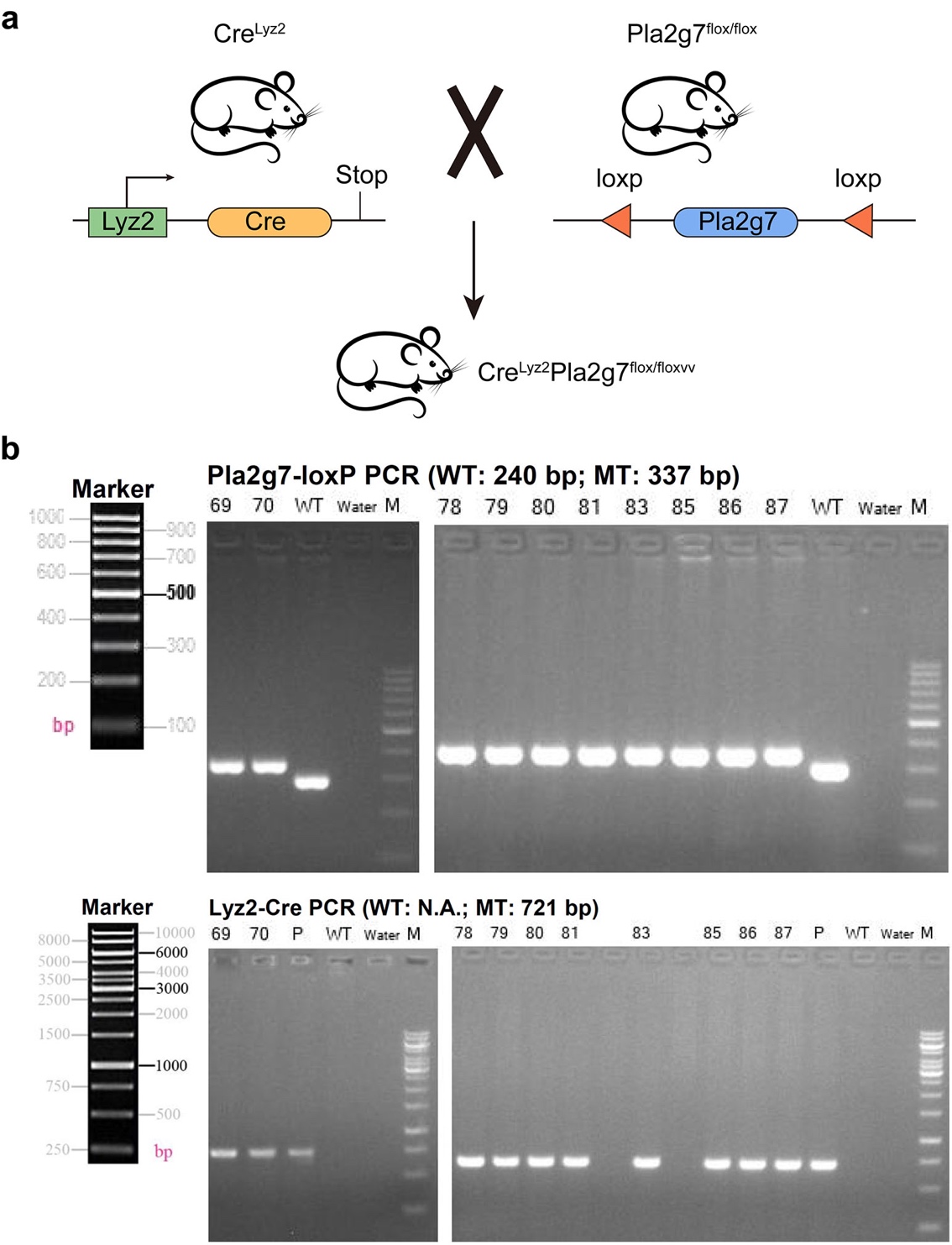
**

**Figure. S6 Generation and validation of Cre^Lyz2^Pla2g7^flox/flox^ mice.**

**
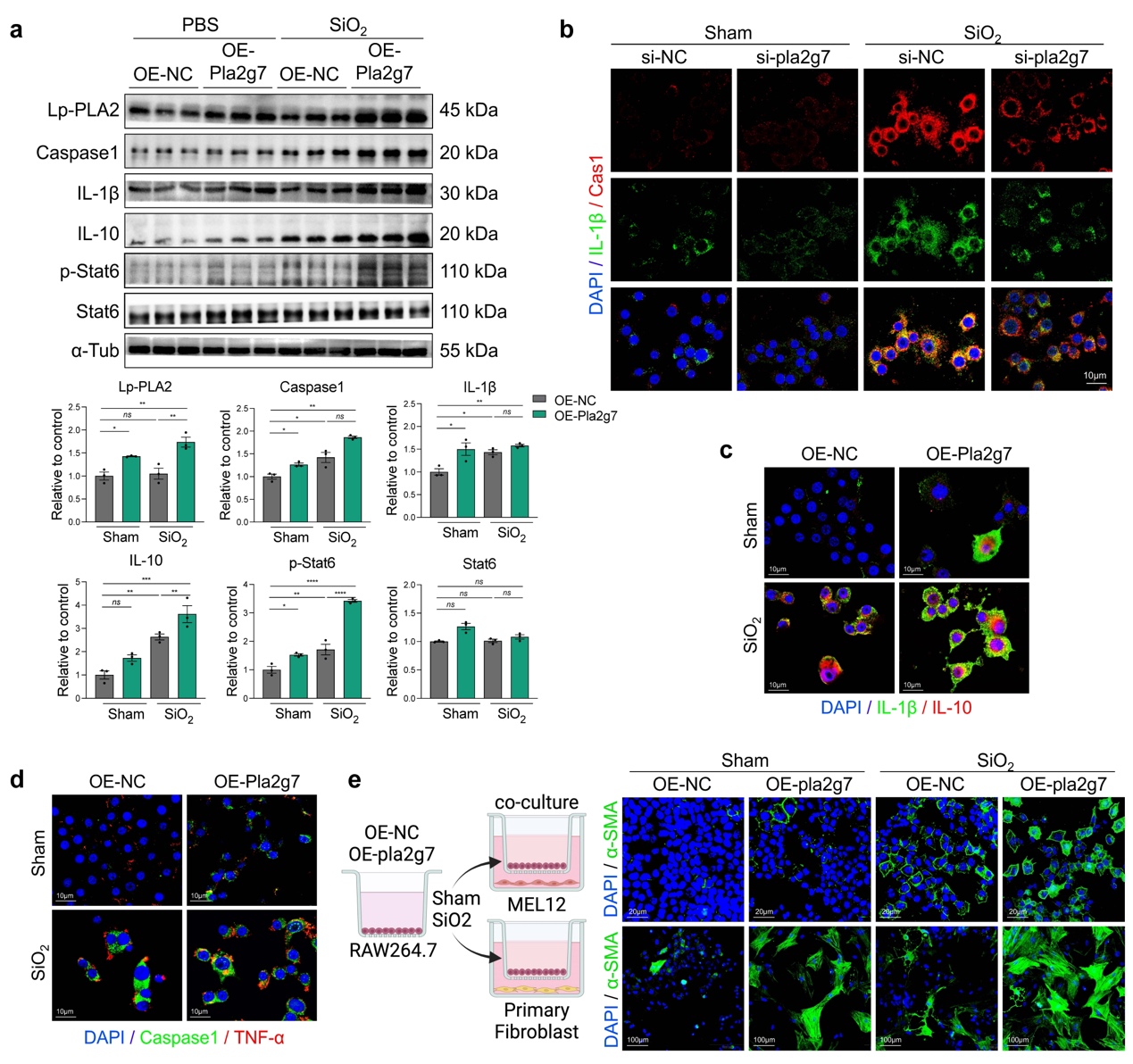
**

**Figure. S7. Effect of Pla2g7 on M1 and M2 polarization in SiO_2_-induced RAW264.7.**

**
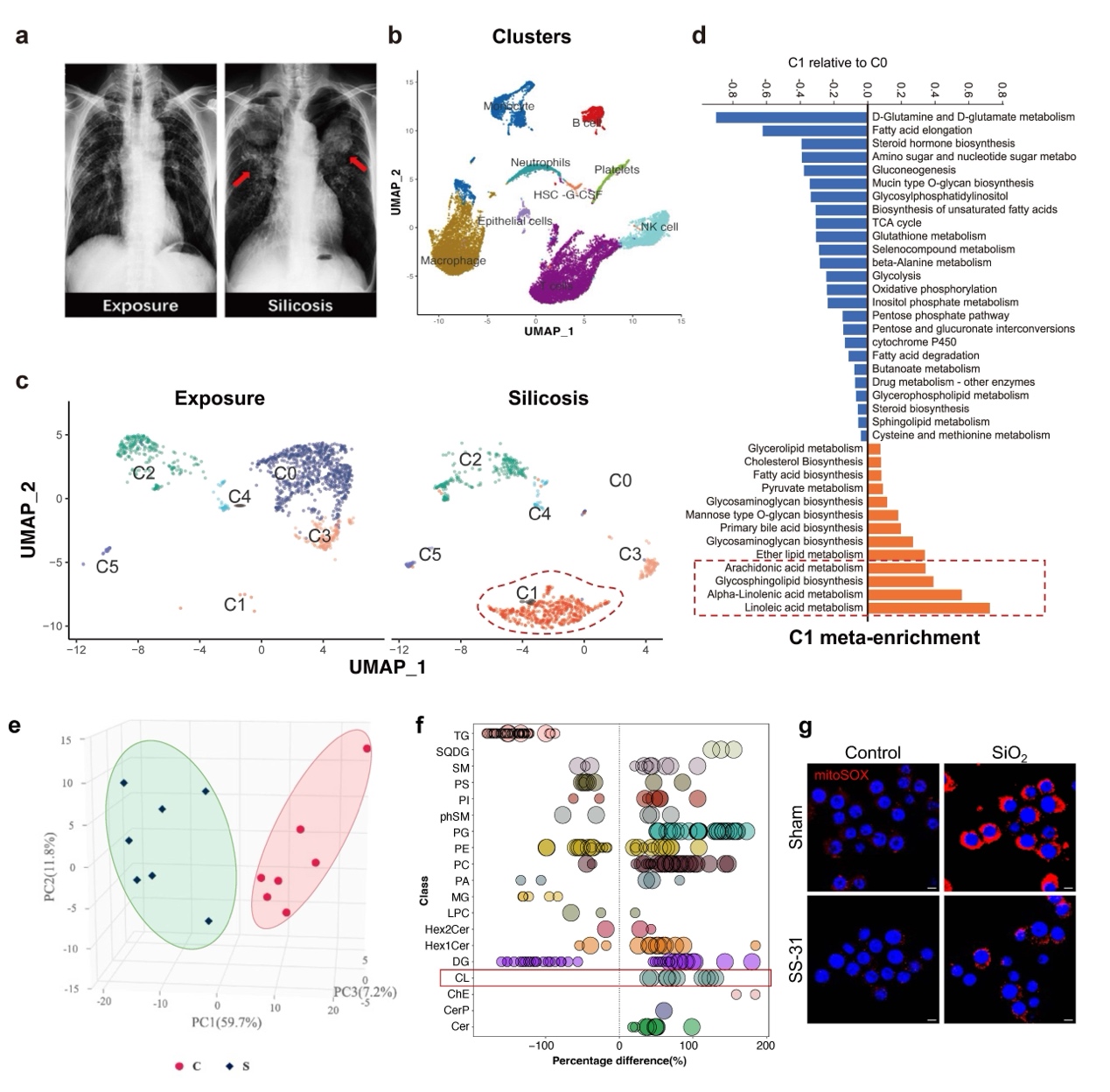
**

**Figure S8 Metabolic pathway analysis macrophage in bronchial alveolar lavage fluid (BALF) of pneumoconiosis patients (GSE174725) and mouse lung tissue.**

**
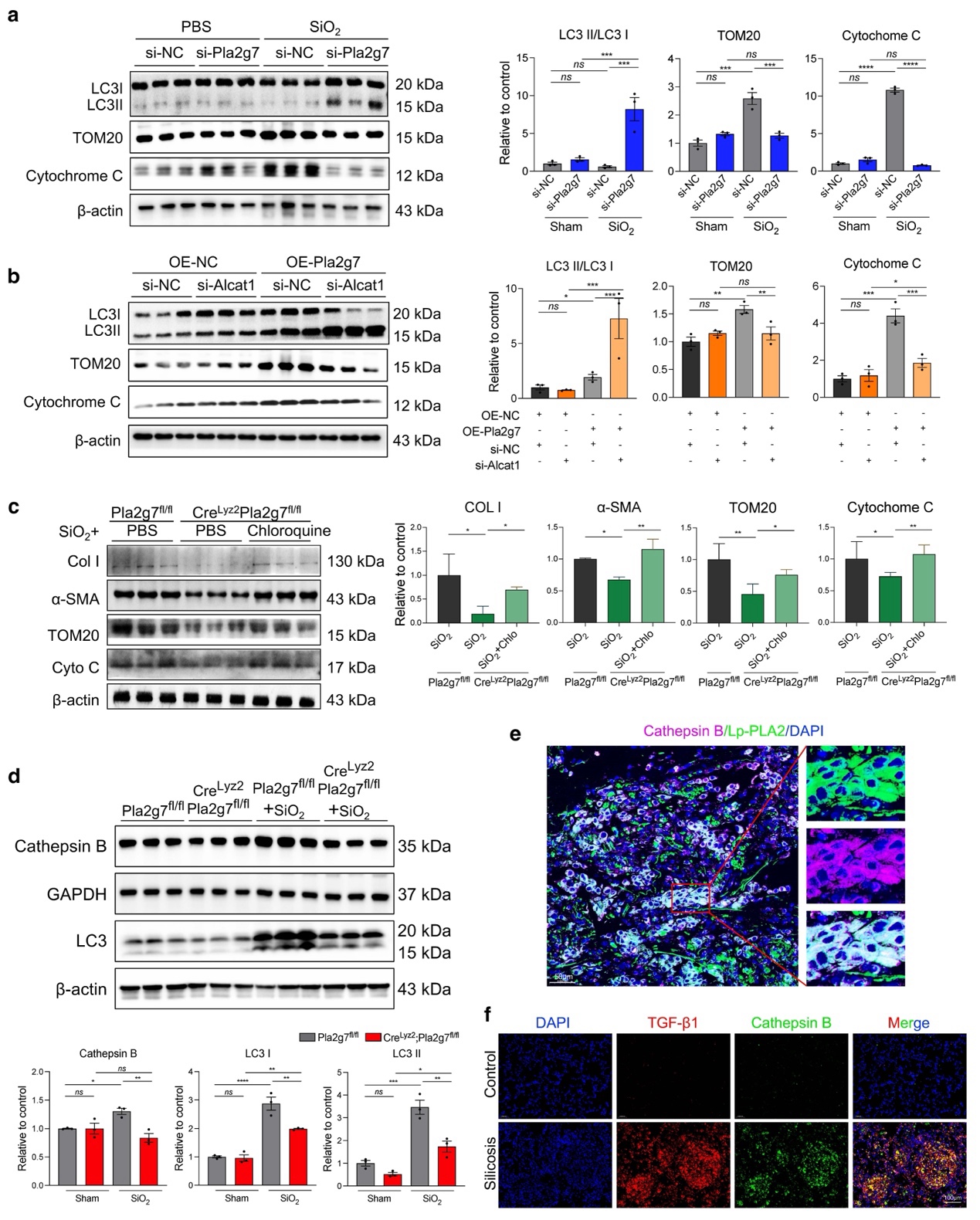
**

**Figure 9 Regulation of the Lp-PLA2-CL pathway on mitophagy and cathepsin B-TGF-β1 signaling in RAW264.7 cells and mouse lung tissue.**

**
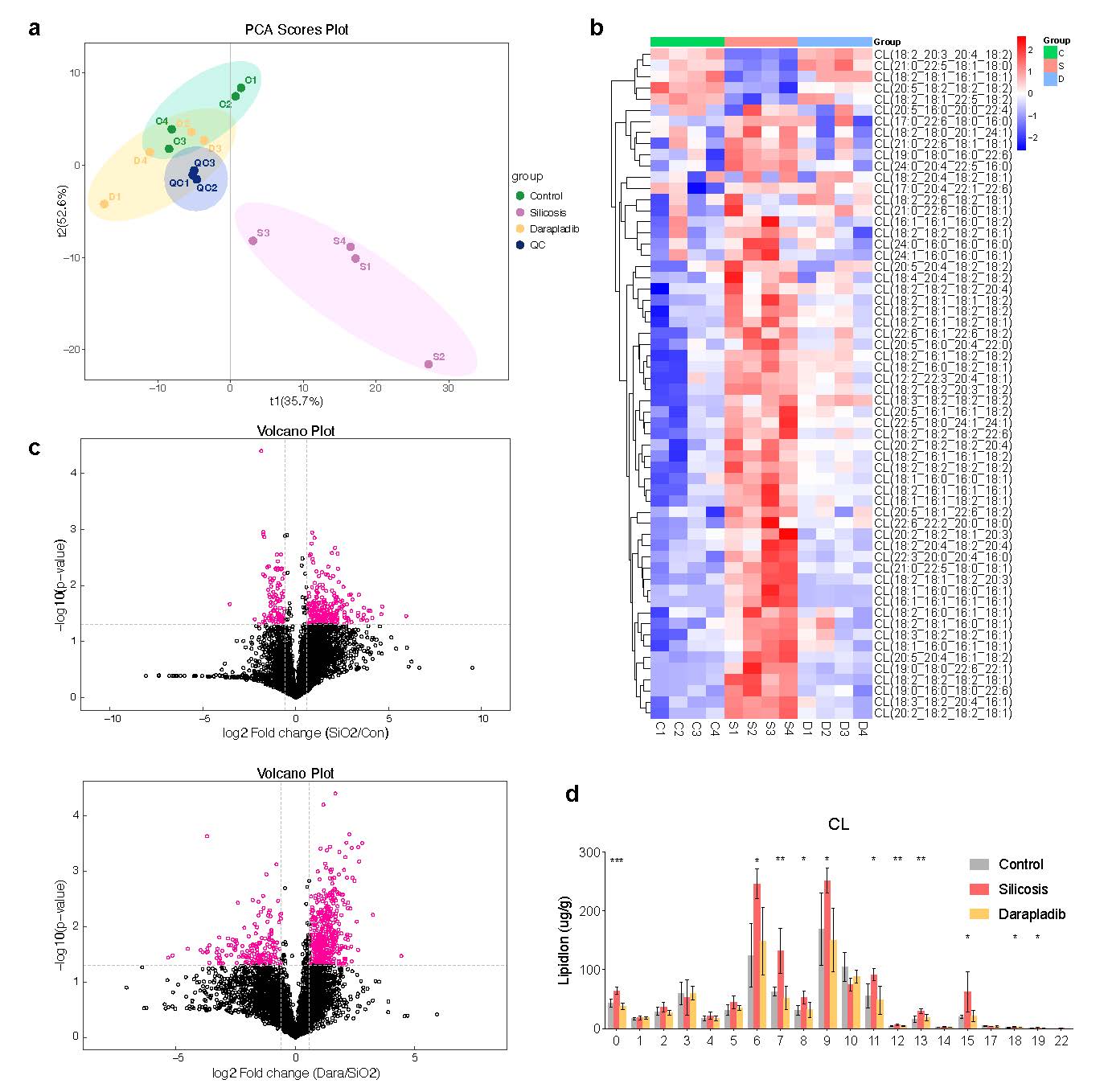
**

**Figure S10 Regulation of the Lp-PLA2-CL pathway on cathepsin B-TGF-β1 signaling in RAW264.7 cells and mouse lung tissue.**

**Table. S1** **Materials and Methods**

| **Reagents** | **Source** | **Identifier** |
| --- | --- | --- |
| **Antibodies** | | |
| APC/Cyanine7 anti-mouse CD45 | BioLegend | 103115 |
| PE anti-mouse Ly-6G | BioLegend | 127607 |
| Brilliant Violet 421™ anti-mouse CD170 (Siglec-F) | BioLegend | 155509 |
| APC anti-mouse CD11c | BioLegend | 117309 |
| PE/Cyanine7 anti-mouse F4/80 | BioLegend | 123113 |
| Alexa Fluor® 488 anti-mouse Ly-6C | BioLegend | 128021 |
| TruStain FcX™ PLUS (anti-mouse CD16/32) | BioLegend | 156603 |
| Zombie Aqua™ Fixable Viability Kit | BioLegend | 423101 |
| Brilliant Violet 650™ anti-mouse/human CD11b | BioLegend | 101239 |
| Alexa Fluor® 488 anti-mouse CX3CR1 Antibody | BioLegend | 149021 |
| Alexa Fluor® 488 Anti-CD68 antibody |  | ab201844 |
| Cd68 | SantaCruz | sc-20060 |
| Purified Rat Anti-Mouse Siglec-F | BD Pharmingen™ | 552125 |
| Anti-Pla2g7 (Lp-PLA2, PAFAH) | Proteintech | Cat No. 15526-1-AP |
| Col I | Proteintech | Cat No. 14695-1-AP |
| α-SMA | Proteintech | Cat No. 14395-1-AP |
| TNF-α | Proteintech | Cat No. 60291-1-Ig |
| Arginase-1 | Proteintech | Cat No : 66129-1-Ig |
| iNOS | Proteintech | Cat No. 22226-1-AP |
| IL-1β | Proteintech | Cat No. 16806-1-AP |
| IL-10 | Proteintech | Cat No. 60269-1-Ig |
| TOM20 | Proteintech | Cat No. 11802-1-AP |
| TOM20 | Proteintech | Cat No. 66777-1-Ig |
| Caspase1 | Proteintech | Cat No. 22915-1-AP |
| ALCAT1 (LCLAT1) | Biorbyt | orb1879621 |
| COX IV | Proteintech | Cat No. 66110-1-Ig |
| Cytochrome C | Proteintech | Cat No. 66264-1-Ig |
| Cathepsin B | Proteintech | Cat No. 12216-1-AP |
| Pink1 | Proteintech | Cat No. 23274-1-AP |
| NLRP3 | Proteintech | Cat No. 68102-1-Ig |
| GSDMD | Abcam | ab209845 |
| IL-6 | Huabio | Catalog# EM1701-45 |
| LC3B | Abcam | ab192890 |
| LC3 | Proteintech | Cat No. 14600-1-AP |
| Ubiquitin | Abcam | ab140601 |
| GAPDH | Proteintech | Cat No. 10494-1-AP |
| β-Actin | Proteintech | Cat No. 81115-1-RR |
| Mouse IL-6 ELISA Kit | BLUE GENE | E03I0006 |
| Mouse IL-1β ELISA Kit | BLUE GENE | E03I0010 |
| Mouse IL-10 ELISA Kit | BLUE GENE | E03I0023 |
| Mouse TGF-β1 ELISA Kit | BLUE GENE | E03T0009 |
| Donkey Anti-Mouse IgG H&L (Alexa Fluor® 555) | Beyotime | A0460 |
| Goat Anti-Rabbit IgG H&L (Alexa Fluor® 488) | Beyotime | A0423 |
| FlexAble CoraLite® Plus 488 Antibody Labeling Kit for Rabbit IgG | Proteintech | Cat No. KFA001 |
| FlexAble CoraLite® Plus 555 Antibody Labeling Kit for Rabbit IgG | Proteintech | Cat No. KFA002 |
| FlexAble CoraLite® Plus 647 Antibody Labeling Kit for Rabbit IgG | Proteintech | Cat No. KFA003 |
| **Chemicals, Peptides, and Recombinant Proteins** | | |
| Cell Staining Buffer | BioLegend | 420201 |
| Fixation Buffer | BioLegend | 420801 |
| Hematoxylin | Solarbio | G1080 |
| Eosin Y solution (Water Soluble) | Solarbio | G1100 |
| Masson | Solarbio | G1340 |
| mitoSOX detection kit | Beyotime | S0033M |
| Mito-Tracker Deep Red 633 | Beyotime | C1034 |
| Darapladib | Targetmol | T6109 |
| Enhanced Chemiluminescent NcmECL High | NCM Biotech | P2300 |
| SiO_2_ | Sigma | s5631 |
| RAW264.7 respective specialized culture medium | Pricella | CM-0190 |
| MLE12 respective specialized culture medium | Pricella | CM-0680 |
| PageRuler Prestained Protein Ladder | Thermo Scientific™ | 26616 |
| Protease inhibitor | Sigma | P8340 |

**Table. S2 Primers used in this study.**

| **Name** | **Sequence (5’**>**3’)** |
| --- | --- |
| PLA2G7-Human | Forward: TCATCAGCATGGGTCAACAAAA Reverse: CCAAAGGGTGTCAAGGCGAT |
| SPP1-Human | Forward: CTCCATTGACTCGAACGACTC Reverse: CAGGTCTGCGAAACTTCTTAGAT |
| GPNMB-Human | Forward: AAGATTGCCACTTGATGCCG Reverse: TCCCTCATGTAAGCAGAAGGTC |
| CD68-Human | Forward: GGAAATGCCACGGTTCATCCA Reverse: TGGGGTTCAGTACAGAGATGC |
| HMOX1-Human | Forward: AAGACTGCGTTCCTGCTCAAC Reverse: AAAGCCCTACAGCAACTGTCG |
| SLPI-Human | Forward: GAGATGTTGTCCTGACACTTGTG Reverse: AGGCTTCCTCCTTGTTGGGT |
| CTSB-Human | Forward: GAGCTGGTCAACTATGTCAACA Reverse: GCTCATGTCCACGTTGTAGAAGT |
| CTSD-Human | Forward: TGCTCAAGAACTACATGGACGC Reverse: CGAAGACGACTGTGAAGCACT |
| TNFAIP2-Human | Forward: GGCCAATGTGAGGGAGTTGAT Reverse: CCCGCTTTATCTGTGAGCCC |
| PSAP-Human | Forward: CCAGCACGACCCAGATTGAC Reverse: AACCCGGCTATCAAACTGAGC |
| LGALS3-Human | Forward: ATGGCAGACAATTTTTCGCTCC Reverse: GCCTGTCCAGGATAAGCCC |
| GAPDH-Human | Forward: GGAGCGAGATCCCTCCAAAAT Reverse: GGCTGTTGTCATACTTCTCATGG |
| Pla2g7-Mouse | Forward: TCACAAGCTCCAATCGGTGAT Reverse: CGACGGGGTACGATCCATTTC |
| Spp1-Mouse | Forward: ATCTCACCATTCGGATGAGTCT Reverse: TGTAGGGACGATTGGAGTGAAA |
| Gpnmb-Mouse | Forward: TGCCAAGCGATTTCGTGATGT Reverse: GCCACGTAATTGGTTGTGCTC |
| Cd68-Mouse | Forward: TGTCTGATCTTGCTAGGACCG Reverse: GAGAGTAACGGCCTTTTTGTGA |
| Hmox1-Mouse | Forward: AGGTACACATCCAAGCCGAGA Reverse: CATCACCAGCTTAAAGCCTTCT |
| Slpi-Mouse | Forward: GGCCTTTTACCTTTCACGGTG Reverse: GGCTCCGATTTTGATAGCATCAT |
| Ctsb-Mouse | Forward: CAGGCTGGACGCAACTTCTAC Reverse: TCACCGAACGCAACCCTTC |
| Ctsd-Mouse | Forward: GCTTCCGGTCTTTGACAACCT Reverse: CACCAAGCATTAGTTCTCCTCC |
| Tnfaip2-Mouse | Forward: AGGAGGAGTCTGCGAAGAAGA Reverse: GGCAGTGGACCATCTAACTCG |
| Pasp-Mouse | Forward: TGATGGCAACCGGGTGTTC Reverse: CCACCACCCTGTTGCTGTAG |
| Lgals3-Mouse | Forward: GGAGAGGGAATGATGTTGCCT Reverse: TCCTGCTTCGTGTTACACACA |
| Mmp12-Mouse | Forward: GGGCTGCTCCCATGAATGAC Reverse: CCAGAGTTGAGTTGTCCAGTTG |
| Il7r-Mouse | Forward: GCGGACGATCACTCCTTCTG Reverse: AGCCCCACATATTTGAAATTCCA |
| Il1rn-Mouse | Forward: TAGACATGGTGCCTATTGACCT Reverse: TCGTGACTATAAGGGGCTCTTC |
| Gapdh-Mouse | Forward: AGGTCGGTGTGAACGGATTTG Reverse: GGGGTCGTTGATGGCAACA |
